# Design, synthesis, and herbicidal activity of indole-3-carboxylic acid derivatives as potential transport inhibitor response 1 antagonists

**DOI:** 10.3389/fchem.2022.975267

**Published:** 2022-07-26

**Authors:** Xing Wang, Mu-Jia Luo, Yu-Xuan Wang, Wen-Qing Han, Jian-Xin Miu, Xi-Ping Luo, Ai-Dong Zhang, Yi Kuang

**Affiliations:** ^1^ Zhejiang Provincial Key Laboratory of Chemical Utilization of Forestry Biomass, College of Chemistry and Materials Engineering, Zhejiang A & F University, Hangzhou, China; ^2^ Key Laboratory of Organic Chemistry of Jiangxi Province, Jiangxi Science & Technology Normal University, Nanchang, China; ^3^ Key Laboratory of Pesticide & Chemical Biology of the Ministry of Education, College of Chemistry, Central China Normal University, Wuhan, China

**Keywords:** herbicide, auxin, indole-3-carboxylic acid (IAA), transport inhibitor response 1 (TIR1), herbicidal activity, molecular docking

## Abstract

Auxins as an important class of phytohormones play essential roles in plant life cycle; therefore, developing compounds with auxin-like properties for plant growth regulation and weed control applications is of great significance. Herein, we reported the design, synthesis, and herbicidal activity evaluation of a series of novel indole-3-carboxylic acid derivatives as auxin receptor protein TIR1 antagonists. Petri dish herbicidal activity assay demonstrated that most of the as-synthesized target compounds exhibited good-to-excellent inhibition effects (60–97% inhibitory rates) on roots and shoots of both dicotyledonous rape (*B. napus*) and monocotyledonous barnyard grass (*E. crus-galli*). The inhibition rates of compounds **10d** and **10h** reached up to 96% and 95% for the root of rape (*B. napus*) at 100 mg/L, and they also maintained 92% and 93% inhibition rates even if at 10 mg/L, respectively. Molecular docking revealed that the interactions between these synthesized target compounds and TIR1 protein include tight π–π stacking, hydrogen bond, and hydrophobic interactions. This work expands the range of auxin chemistry for the development of new auxin mimic herbicides.

## 1 Introduction

Sustainable technologies that increase the yield of crop and lower the impact on environment are critically important to meet the growth and demographic changes of population. One of the keys to implement agriculture intensification is to minimize crop losses due to competition from weeds with crops for water, light, and nutrients (Rauzan and Lorsbach, 2021). Weeds can be controlled by physical, biological, mechanical, or chemical methods, and chemical control is the most efficient. Herbicides as weed control agrochemicals play an irreplaceable role in achieving increase in crop production and ensuring national food security (Gianessi, 2013), and they were considered as a convenient, effective, and powerful tool to interrupt or destroy the growth of weeds in agricultural fields (Moss, 2019). However, with the extensive and frequent use of chemical herbicides, weed resistance and environment hazard are dramatically increased over the world (Tudi et al., 2021; Heap, 2022). Therefore, sustainability in agriculture calls for designing sustainable herbicides with high efficacy, low toxicity, low-persistent, low-level resistance, and environmental-friendly properties, which as parts of the 2030 sustainability goals set by Corteva Agriscience (sustainability, 2021). Designing small molecules that mimic naturally occurring molecules in the plants provides an opportunity to deliver sustainable herbicide active compounds. Natural auxins are an important class of phytohormones which regulate cell division, elongation, and developmental processes, including vascular tissue and floral meristem differentiation, leaf initiation, senescence, and apical dominance (Grossmann, 2010). Indole-3-acetic acid (IAA) is one of the natural auxins and considered as a “master hormone” in the network of interactions with other phytohormones regulating plant growth and development (Bauer et al., 2018). The cellular concentration and stability of the natural auxin IAA are controlled by multiple pathways such as biosynthesis, transport, localization, derivatization, and degradation (Zhao, 2010). On the contrary, synthetic auxin herbicides are relatively long-lived xenobiotic compounds which are not controlled by these complex homeostatic mechanisms that keep endogenous IAA levels in balance. Therefore, synthetic auxin mimics have found practical use not only as plant growth regulators but also as herbicides to control weeds (Ross et al., 2001; Grossmann 2003; Pieterse et al., 2012; Tan et al., 2012; Agehara and Leskovar, 2015; Ebrahimzadeh et al., 2018; Tung et al., 2018).

**FIGURE 1:**
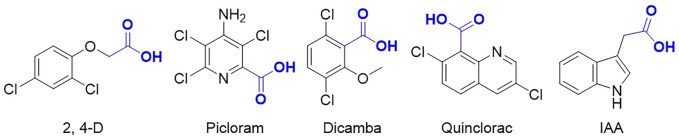
Chemical structures of typical auxinic herbicides and indole-3-acetic acid (IAA).

Auxinic herbicides, which include four groups such as phenoxyalkanoic acid (2,4-D, 2,4-DP, 2,4-DB, 2,4,5-T, MCPA, and MCPB), pyridinecarboxylic acid (fluroxypyr, triclopyr, clopyralid, and picloram), benzoic acid (dicamba), and quinoline acid (quinmerac and quinclorac) (Figure 1), are widely used in agriculture to selectively control broadleaf weeds and revolutionized modern agricultural production throughout the world (Marumo et al., 1971; Bandurski et al., 1995; Indegit and Duke, 2003; Verma et al., 2009; Mithila et al., 2011; Du et al., 2014; Jeschke et al., 2019). Due to their absolute advantages such as selectivity, efficacy, wide spectrum of weed control, and low application costs (Dharmasiri et al., 2005), these herbicides have been a preferred choice for weed control and are the most extensively used herbicides worldwide for more than 80 years since 2,4-D was developed in 1945. Auxinic herbicides are structurally similar to the natural plant hormone auxin, indole-3-acetic acid (IAA), and induce several of the same physiological and biochemical responses at high concentrations but are more durable and effective because they are not rendered inactive by the plant as rapidly as the endogenous auxins.

The receptor for auxin was identified in recent studies as transport inhibitor response 1 (TIR1) protein, which is an F-box protein component of an SCF (SKP1, Cullin1, F-box)-type E3 ubiquitin ligase complex and responsible for tagging Aux/IAA proteins for proteolysis by 26S proteasomes (Leyser, 2002; Kepinski and Leyser, 2005; Hayashi, 2012; Korasick et al., 2015; Salehin et al., 2015). In this signaling pathway, auxinic herbicide molecule binds to auxin receptor TIR1, which facilitates the interactions between TIR1 and co-repressor Aux/IAA proteins, leading to the ubiquitination and degradation of the Aux/IAA proteins. Consequently, the expression of auxin responsive genes is elevated, which leads to the death of weeds (Enders and Strader, 2015; Cao et al., 2017; Myo et al., 2019; Duca and Glick, 2020). Thus, the chemical manipulation of the auxin-signaling pathway via synthetic auxin mimics for developing herbicides has gained enormous interest.

**FIGURE 2:**
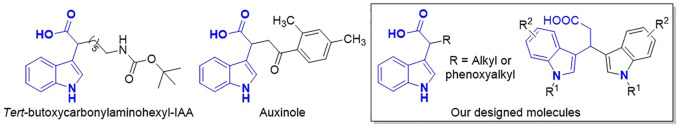
Reported structures of TIR1 antagonists and our designed molecules

Recently, a lot of auxin mimic herbicides have been uncovered with greater herbicidal activities on a broader spectrum of weeds at lower use rates (Bell et al., 2019). These newer herbicides generally consist of a planar pyridine or pyrimidine group with an attached carboxylic acid functionality and act as TIR1 agonists. However, there are few reports on anti-auxinic compounds. Hayashi et al. reported two IAA analogs, *tert*-butoxycarbonylaminohexyl-IAA and auxinole (Figure 2), which exhibited potent antagonistic activity by blocking the formation of the TIR1-IAA-Aux/IAA complex and inhibited auxin-responsive gene expression in plants (Hayashi et al., 2008; Hayashi et al., 2012). Molecular docking analysis indicated that the phenyl ring in auxinole would strongly interact with Phe82 of TIR1, a residue that is crucial for Aux/IAA recognition. Related work provided a new method for developing TIR1 antagonists as novel auxinic herbicides for weed control.

In order to discover new TIR1 antagonists as auxinic herbicides, a total of 24 novel α-substituted indole-3-carboxylic acid derivatives were designed, synthesized, and characterized in this study. These compounds were assessed under incubator conditions to determine their herbicidal activities against monocotyledon and dicotyledon weeds, and their structure–activity relationships were studied.

## 2 Materials and methods

### 2.1 General

^1^H NMR spectra were recorded using 400/600 MHz spectrometers. Chemical shifts (*δ*) were reported in ppm quoted relative to internal tetramethylsilane (internal standard, 0.0 ppm) with the coupling constants (*J*) given in Hz. ^13^C NMR spectra were recorded with the same spectrometer operating at 100/150 MHz with complete proton decoupling (internal standard CDCl_3_: 77.0 ppm, DMSO-*d*_*6*_: 39.5 ppm). Splitting patterns were assigned, s = singlet, d = doublet, t = triplet, dd = double doublet, etc. Mass spectra were measured with a Finnigan Trace MS spectrometer. IR measurements were performed on Bruker TENSOR27 using KBr as the substrate. Elementary analysis was taken with a Vario EL III elemental analysis instrument. Melting points (m.p.) were obtained on a digital melting point apparatus (Biddy-Electrothermal) without correction. Unless otherwise noted, materials were used as commercially supplied. All reactions were monitored by TLC analysis on silica gel–coated plates. Flash column chromatography was performed by using 200–300 mesh silica gel.

### 2.2 Chemical synthesis

**FIGURE 3:**
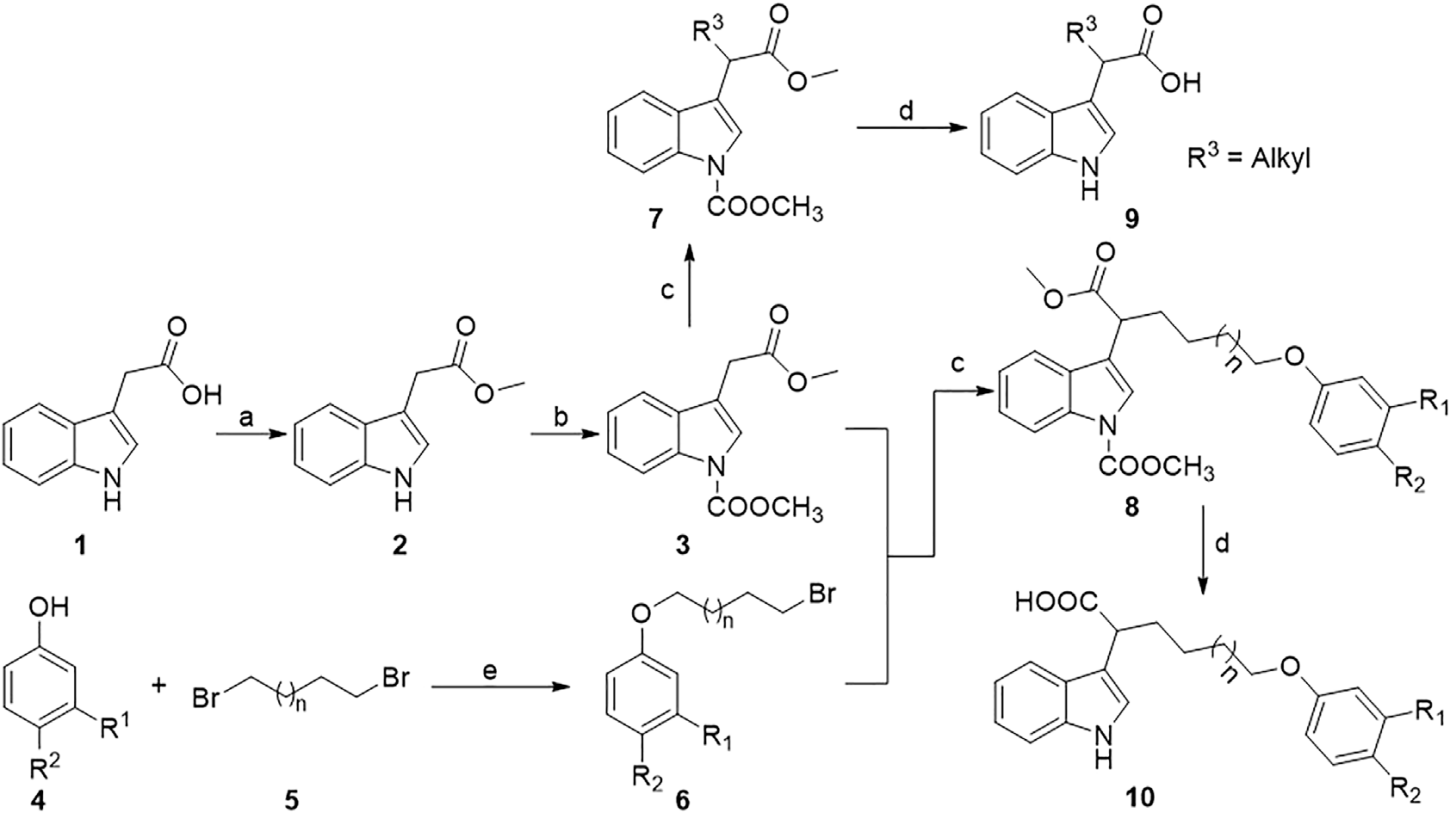
Synthetic route of α-substituted indole-3-acetic acids. Reagents and conditions: a) DCC, DMAP, MeOH, dry CH_2_Cl_2_, r.t.; b) TEBAC, methyl chloroformate, DCM, 30% NaOH (*aq.*), ice bath; c) R-Br, LDA, HMPA, THF, −78°C–0°C; d) 30% NaOH (*aq.*): CH_3_OH = 1:2 (v/v), 70°C, 4 h, 2 M HCl.

**FIGURE 4:**
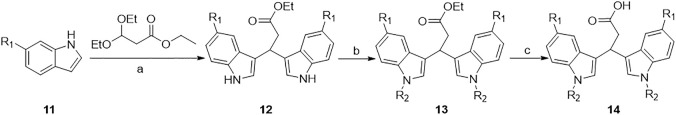
Synthetic route of 3,3-diindolepropionic acid derivatives. Reagents and conditions: a) CH_3_COOH, KHSO_4_ < 80°C, 5 h; b) KOH, R-X, THF, r.t.; c) LiOH, CH_3_OH: H_2_O = 1:1 (v/v), 75°C, 2 h, 2 M HCl.

All the indole-3-carboxylic acid derivatives included in this report were synthesized using the sequences shown in Figure 3 and Figure 4. As shown in Figure 3, protection of indole-3-acetic acid in the presence of methanol and methyl chloroformate allowed the formation of the intermediate **3**, which undergoes nucleophilic substitution reaction with alkyl bromide under strong base lithium diisopropylamide (LDA) conditions to give intermediates **7** and **8**. Finally, deprotection of intermediates **7** and **8** in the presence of 30% sodium hydroxide aqueous solution allowed the formation of the target compounds **9** and **10**. The 3,3-diindolepropionic acid compounds **12** can be conveniently obtained by a potassium bisulfate catalyzed one-pot reaction using substituted indole acetic acid and ethyl 3,3-diethoxypropionate as raw materials. Then, the nucleophilic substitution of compounds **12** with alkyl bromide produces compounds **13**, which undergoes hydrolyzation under lithium hydroxide and methanol conditions to give compounds **14** (Figure 4).

**Synthesis of intermediate 2:** indole-3-acetic acid (10.0 mmol, 1.75 g), dicyclohexylcarbodiimide (DCC, 2.06 g, 10.0 mmol), 4-dimethylaminopyridine (DMAP, 1.0 mmol, 0.13 g), anhydrous dichloromethane (40 ml), and methanol (1 ml) were successively added into a 100-ml round-bottom flask at room temperature. The mixture was stirred for 4 h and monitored using thin-layer chromatography (TLC). When the reaction was completed, the resulting dicyclohexylurea was filtered out and 20 ml water was added into the filtrate, and extracted with dichloromethane for 3 times (3 × 50 ml). The combined organic phase was washed with saturated saline solution (3 × 100 ml), dried with anhydrous Na_2_SO_4_, and concentrated in vacuo to give a residue. The residue was purified by column chromatography on silica gel using 10:1 (v/v) petroleum ether/ethyl acetate mixtures to obtain intermediate **2** as faint yellow oil in 79% yield.

**Synthesis of intermediate 3:** methyl indole-3-acetate (10.0 mmol, 1.89 g), benzyltriethylammonium chloride (0.05 mmol, 0.11 g), dichloromethane (15 ml), and 30% NaOH aqueous solution (15 ml) were successively added into a 50-ml round-bottom flask under an ice-bath condition. Then, methyl chloroformate (15 mmol, 1.43 g) was slowly added dropwise. After that, the reaction mixture was gradually warmed up to room temperature and stirred for 2 h. Then, the typical treating process as aforementioned for intermediate **2** was followed to obtain intermediate **3** as yellow oil in 85% yield.

**Synthesis of intermediate 6:** 1, 4-dibromobutane (10 mmol, 2.16 g) or 1,5-dibromopentane (10 mmol, 2.30 g), cesium carbonate (10 mmol, 3.25 g), and 25 ml acetonitrile were added into a 50-ml round-bottom flask. The mixture was stirred at 85°C, and then, a solution of substituted phenol (5 mmol, 0.5 equiv.) in 5 ml acetonitrile was slowly added dropwise. After that, the reaction mixture was stirred for 3 h and monitored by TLC. When the reaction was finished, 20 ml water was added and extracted with ethyl acetate for three times (3 × 50 ml). Then, the typical purification process as aforementioned was carried out to obtain intermediate **6**.

**Synthesis of intermediates 7 and 8:** the solution of redistilled diisopropylamine (3.0 mmol, 0.43 ml) in 5 ml tetrahydrofuran (THF) was added into a 50-ml dry three-neck flask under argon atmosphere, the flask was placed in a −78 °C constant temperature reactor, and then *n*-butyllithium (3 mmol, 1.3 ml) was slowly added dropwise into the mixture and stirred for 1 h. After that, hexamethylphosphoric triamide (HMPA, 14.65 mmol, 2.63 g) was added and stirred for another 20 min, and then, a solution of intermediate **3** (2.0 mmol, 0.49 g) in 5 ml THF was added and continued to stir for 30 min. Finally, the solution of alkyl bromide or intermediate **6** (3 mmol, 1.5 equiv.) in THF (5 ml) was added and stirred for another 1 h. Then, the reaction mixture was gradually heated to 0°C and monitored by TLC. When the reaction was completed, 20 ml water was added to quench the reaction and extracted with ethyl acetate for three times (3 × 50 ml). Then, the typical purification process as aforementioned was carried out to obtain intermediates **7** and **8**.

**Synthesis of target compounds 9 and 10:** intermediate **7** or **8** (1 mmol) was dissolved in 10 ml mixed solution of MeOH and 2 M NaOH (v/v, 4:1), and the mixture was stirred and reacted at 75°C for 4 h. After that, the reaction mixture was cooled to room temperature, acidified with 2 M hydrochloric acid to pH = 3, and extracted with ethyl acetate for three times (3 × 30 ml). Then, the typical purification process as aforementioned was carried out to produce the target compounds **9** in 71–78% yields and **10** in 54–68% yields, respectively.

### 2.3 2-(1*H*-indol-3-yl)hexanoic acid (9a)

As a rufous oil in 71% yield. ^1^H NMR (600 MHz, CDCl_3_): *δ* 10.77 (s, 1H), 7.79 (s, 1H), 7.66 (d, *J* = 7.7 Hz, 1H), 7.16–7.02 (m, 3H), 6.77 (s, 1H), 3.80 (t, *J* = 7.5 Hz, 1H), 2.14–2.03 (m, 1H), 1.90–1.79 (m, 1H), 1.28–1.24 (m, 4H), 0.80 (d, *J* = 6.1 Hz, 3H); ^13^C NMR (150 MHz, CDCl_3_): *δ* 181.31, 135.93, 126.28, 122.39, 121.91, 119.43, 118.97, 112.73, 111.31, 42.85, 31.94, 29.67, 22.32, 13.76; IR (KBr, cm^−1^): 3417, 3061, 2961, 2870, 1710, 1461, 1417, 1353, 1212, 1104, 947, 739; EI-MS m/z: 231.14 (M^+^, 58.63), 130.04(100); Elem. Anal. Calcd. for C_14_H_17_NO_2_: C, 72.70; H, 7.41; N, 6.06. Found: C, 72.95; H, 7.35; N, 6.22.

### 2.4 2-(1*H*-indol-3-yl)decanoic acid (9b)

As a rufous oil in 73% yield. ^1^H NMR (600 MHz, DMSO): *δ* 12.10 (s, 1H), 10.93 (s, 1H), 7.57 (d, *J* = 7.9 Hz, 1H), 7.33 (d, *J* = 8.2 Hz, 1H), 7.19 (s, 1H), 7.06 (t, *J* = 7.6 Hz, 1H), 6.96 (t, *J* = 7.4 Hz, 1H), 3.69 (t, *J* = 7.6 Hz, 1H), 2.05–1.96 (m, 1H), 1.80–1.72 (m, 1H), 1.27–1.21 (m, 12H), 0.84 (t, *J* = 6.8 Hz, 3H); ^13^C NMR (150 MHz, CDCl_3_): *δ*181.33, 136.02, 126.42, 122.27, 122.07, 119.55, 119.15, 113.15, 111.24, 42.93, 32.35, 31.79, 29.36, 29.33, 29.21, 27.64, 22.60, 14.07; IR (KBr, cm^−1^): 3417, 3068, 2928, 2860, 1710, 1454, 1353, 1296, 1220, 1104, 947, 741; EI-MS m/z: 287.32 (M^+^, 32.10), 130.07(100); Elem. Anal. Calcd. for C_18_H_25_NO_2_: C, 75.22; H, 8.77; N, 4.87. Found: C, 75.56; H, 8.94; N, 4.84.

### 2.5 2-(1*H*-indol-3-yl)tetradecanoic acid (9c)

As a pale yellow solid in 78% yield, m.p. 72.7–73.6°C. ^1^H NMR (600 MHz, DMSO): *δ* 11.80 (s, 1H), 8.03 (d, *J* = 8.1 Hz, 1H), 7.80 (t, *J* = 6.8 Hz, 1H), 7.36 (d, *J* = 7.9 Hz, 1H), 7.30–7.24 (m, 1H), 7.21 (dd, *J* = 13.6, 6.5 Hz, 1H), 7.09 (d, *J* = 10.6 Hz, 1H), 3.95 (d, *J* = 6.9 Hz, 1H), 2.21 (d, *J* = 12.7 Hz, 1H), 2.00 (s, 1H), 1.58–1.17 (m, 20H), 0.98 (t, *J* = 6.9 Hz, 3H).; ^13^C NMR (150 MHz, CDCl_3_): *δ* 181.29, 136.03, 126.46, 122.24, 122.12, 119.59, 119.20, 113.26, 111.22, 42.93, 32.39, 31.90, 29.64, 29.41, 29.34, 27.67, 22.68, 14.12; IR (KBr, cm^−1^): 3424, 3061, 2928, 2852, 1693, 1461, 1421, 1212, 1096, 955, 739; EI-MS m/z: 343.16 (M^+^, 60.74), 130.04(100); Elem. Anal. Calcd. for C_22_H_33_NO_2_: C, 76.92; H, 9.68; N, 4.08. Found: C, 77.37; H, 9.98; N, 4.25.

### 2.6 6-(4-Fluorophenoxy)-2-(1*H*-indol-3-yl)hexanoic acid (10a)

As a rufous oil in 65% yield. ^1^H NMR (600 MHz, DMSO): *δ* 10.18 (s, 1H), 8.21 (s, 1H), 7.85 (d, *J* = 7.9 Hz, 1H), 7.36 (d, *J* = 8.0 Hz, 1H), 7.31 (t, *J* = 7.4 Hz, 1H), 7.25 (t, *J* = 7.4 Hz, 1H), 7.08 (d, *J* = 1.5 Hz, 1H), 7.05 (t, *J* = 8.6 Hz, 2H), 6.97–6.72 (m, 2H), 4.14–3.99 (m, 1H), 3.87 (dt, *J* = 14.8, 7.6 Hz, 2H), 2.44–2.21 (m, 1H), 2.10 (dq, *J* = 9.5, 7.2 Hz, 1H), 1.99–1.77 (m, 2H), 1.71–1.51 (m, 2H); ^13^C NMR (150 MHz, CDCl_3_): *δ* 180.67, 157.75, 156.18, 154.85, 135.99, 126.24, 122.40, 122.06, 119.54, 119.02, 115.65, 115.50, 115.35, 115.30, 112.62, 111.30, 68.10, 42.76, 31.82, 28.80, 24.00; IR (KBr, cm^−1^): 3433, 3078, 2946, 2854, 1693, 1502, 1460, 1302, 1245, 1212, 1104, 1038, 938, 831, 739; EI-MS m/z: 341.25 (M^+^, 79.86), 130.05(100); Elem. Anal. Calcd. for C_20_H_20_FNO_3_: C, 70.37; H, 5.91; N, 4.10. Found: C, 70.51; H, 5.88; N, 4.21.

### 2.7 6-(4-Chlorophenoxy)-2-(1*H*-indol-3-yl)hexanoic acid (10b)

As a pale red solid in 54% yield, m.p. 80.3–81.1°C. ^1^H NMR (400 MHz, DMSO): *δ* 12.08 (s, 1H), 10.90 (s, 1H), 7.56 (d, *J* = 7.9 Hz, 1H), 7.32 (d, *J* = 8.0 Hz, 1H), 7.26 (d, *J* = 8.9 Hz, 2H), 7.19 (d, *J* = 2.4 Hz, 1H), 7.04 (t, *J* = 7.5 Hz, 1H), 6.94 (t, *J* = 7.4 Hz, 2H), 6.88 (d, *J* = 8.9 Hz, 1H), 3.90 (t, *J* = 6.4 Hz, 2H), 3.72 (t, *J* = 7.5 Hz, 1H), 2.06 (s, 1H), 1.86 (s, 1H), 1.78–1.62 (m, 1H), 1.41 (d, *J* = 6.1 Hz, 2H); ^13^C NMR (100 MHz, CDCl_3_): *δ* 185.15, 180.24, 157.18, 135.86, 128.98, 126.15, 125.06, 122.20, 122.01, 119.48, 118.96, 115.56, 112.70, 111.17, 67.84, 42.89, 32.02, 28.92, 24.20; IR (KBr, cm^−1^): 3417, 3061, 2945, 2871, 1701, 1594, 1486, 1287, 1245, 1096, 1006, 822, 748, 673; EI-MS m/z: 357.32 (M^+^, 31.42), 130.08(100); Elem. Anal. Calcd. for C_20_H_20_ClNO_3_: C, 67.13; H, 5.63; N, 3.91. Found: C, 67.06; H, 5.56; N, 3.85.

### 2.8 2-(1*H*-indol-3-yl)-6-(4-nitrophenoxy)hexanoic acid (10c)

As a yellow solid in 59% yield, m.p. 76.8–77.7°C. ^1^H NMR (600 MHz, DMSO): *δ* 12.19 (s, 1H), 10.97 (s, 1H), 8.15 (d, *J* = 6.2 Hz, 2H), 7.63 (d, *J* = 5.3 Hz, 1H), 7.38 (d, *J* = 5.9 Hz, 1H), 7.26 (s, 1H), 7.04 (dd, *J* = 30.5, 24.2 Hz, 4H), 4.04 (s, 2H), 3.78 (s, 1H), 2.12 (s, 1H), 1.94 (d, *J* = 54.9 Hz, 1H), 1.77 (s, 2H), 1.45 (s, 2H); ^13^C NMR (150 MHz, DMSO): *δ* 175.59, 164.01, 140.67, 136.29, 126.43, 125.84, 122.92, 121.06, 118.92, 118.49, 114.90, 112.84, 111.55, 68.54, 42.64, 39.92, 31.88, 28.33, 23.86.; IR (KBr, cm^−1^): 3424, 3078, 2945, 2870, 1701, 1593, 1502, 1345, 1262, 1178, 1104, 1004, 847, 748; EI-MS m/z: 368.04 (M^+^, 11.44), 130.03(100); Elem. Anal. Calcd. for C_20_H_20_N_2_O_5_: C, 65.21; H, 5.47; N, 7.60. Found: C, 65.47; H, 7.51; N, 7.23.

### 2.9 2-(1*H*-indol-3-yl)-6-(3-(trifluoromethyl)phenoxy)hexanoic acid (10d)

As a rufous oil in 58% yield. ^1^H NMR (600 MHz, CDCl_3_): *δ* 10.43 (s, 1H), 7.93 (s, 1H), 7.67 (d, *J* = 7.4 Hz, 1H), 7.22 (t, *J* = 7.5 Hz, 1H), 7.13 (dd, *J* = 14.8, 7.0 Hz, 3H), 7.08–7.01 (m, 2H), 6.89 (d, *J* = 7.7 Hz, 1H), 6.86 (s, 1H), 3.84 (t, *J* = 6.8 Hz, 1H), 3.75 (s, 2H), 2.13 (s, 1H), 1.92 (s, 1H), 1.68 (s, 2H), 1.42 (d, *J* = 5.2 Hz, 2H); ^13^C NMR (150 MHz, CDCl_3_): *δ* 180.81, 158.94, 136.04, 131.79, 131.58, 131.36, 131.15, 129.82, 126.79, 126.73, 126.26, 124.92, 123.12, 122.46, 122.10, 121.31, 119.58, 119.03, 117.85, 117.04, 112.59, 111.35, 111.06, 67.69, 42.82, 31.78, 28.66, 23.98.; IR (KBr, cm^−1^): 3418, 3067, 2944, 2879, 1710, 1610, 1462, 1338, 1237, 1171, 1131, 888, 798, 748; EI-MS m/z: 391.29 (M^+^, 77.63), 130.05(100); Elem. Anal. Calcd. for C_21_H_20_F_3_N_2_O_3_: C, 64.44; H, 5.15; N, 3.58. Found: C, 64.54; H, 4.90; N, 3.86.

### 2.10 6-(4-Fluoro-3-(trifluoromethyl)phenoxy)-2-(1*H*-indol-3-yl)hexanoic acid (10e)

As a rufous oil in 61% yield. ^1^H NMR (600 MHz, DMSO): *δ* 12.15 (s, 1H), 10.95 (s, 1H), 7.59 (d, *J* = 7.9 Hz, 1H), 7.39 (d, *J* = 9.6 Hz, 1H), 7.37–7.32 (m, 1H), 7.22 (d, *J* = 8.5 Hz, 2H), 7.19 (d, *J* = 5.3 Hz, 1H), 7.07 (t, *J* = 7.5 Hz, 1H), 6.97 (t, *J* = 7.4 Hz, 1H), 3.98 (t, *J* = 6.3 Hz, 2H), 3.75 (t, *J* = 7.5 Hz, 1H), 2.21–2.03 (m, 1H), 1.94–1.80 (m, 1H), 1.80–1.65 (m, 2H), 1.42 (d, *J* = 5.9 Hz, 2H); ^13^C NMR (150 MHz, DMSO): *δ* 175.60, 154.87, 153.70, 152.07, 136.32, 126.45, 123.43, 122.93, 121.63, 121.06, 120.34, 118.92, 118.48, 118.18, 118.04, 117.14, 112.86, 112.31, 111.56, 109.63, 68.47, 42.66, 39.92, 31.91, 28.47, 23.92.; IR (KBr, cm^−1^): 3418, 3068, 2945, 2871, 1710, 1444, 1336, 1259, 1220, 1139, 1045, 821, 739; EI-MS m/z: 409.01 (M^+^, 43.54), 130.03(100); Elem. Anal. Calcd. for C_21_H_19_F_4_NO_3_: C, 61.61; H, 4.68; N, 3.42. Found: C, 61.80; H, 4.61; N, 3.34.

### 2.11 7-(4-Fluorophenoxy)-2-(1*H*-indol-3-yl)heptanoic acid (10f)

As a rufous oil in 62% yield. ^1^H NMR (400 MHz, DMSO): *δ* 12.06 (s, 1H), 10.89 (s, 1H), 7.56 (d, *J* = 7.8 Hz, 1H), 7.32 (d, *J* = 8.0 Hz, 1H), 7.19 (d, *J* = 2.3 Hz, 1H), 7.11–7.01 (m, 3H), 6.95 (t, *J* = 7.4 Hz, 1H), 6.90–6.84 (m, 2H), 3.88 (t, *J* = 6.4 Hz, 2H), 3.72 (t, *J* = 7.5 Hz, 1H), 2.05 (dt, *J* = 13.3, 8.2 Hz, 1H), 1.81 (dt, *J* = 13.4, 6.9 Hz, 1H), 1.71–1.60 (m, 2H), 1.43 (dt, *J* = 14.3, 7.3 Hz, 2H), 1.38–1.28 (m, 2H); ^13^C NMR (100 MHz, CDCl_3_): *δ* 185.24, 180.25, 157.97, 155.62, 154.84, 135.90, 126.27, 122.12, 122.05, 119.51, 119.05, 115.65, 115.42, 115.28, 115.20, 113.07, 111.15, 68.38, 42.91, 32.37, 29.09, 27.47, 25.89; IR (KBr, cm^−1^): 3416, 3061, 2946, 2862, 1710, 1626, 1509, 1469, 1212, 1104, 1013, 831, 748; EI-MS m/z: 355.36 (M^+^, 39.08), 130.06(100); Elem. Anal. Calcd. for C_21_H_22_FNO_3_: C, 70.97; H, 6.24; N, 3.94. Found: C, 70.80; H, 6.32; N, 4.19.

### 2.12 2-(1*H*-indol-3-yl)-7-(4-nitrophenoxy)heptanoic acid (10 g)

As a yellow oil in 67% yield. ^1^H NMR (600 MHz, DMSO): *δ* 12.11 (s, 1H), 10.93 (s, 1H), 8.18 (d, *J* = 8.9 Hz, 2H), 7.58 (d, *J* = 7.8 Hz, 1H), 7.34 (d, *J* = 8.0 Hz, 1H), 7.21 (s, 1H), 7.10 (d, *J* = 8.9 Hz, 2H), 7.06 (t, *J* = 7.4 Hz, 1H), 6.96 (t, *J* = 7.3 Hz, 1H), 4.08 (t, *J* = 6.0 Hz, 2H), 3.72 (s, 1H), 2.04 (s, 1H), 1.81 (s, 1H), 1.71 (d, *J* = 6.7 Hz, 2H), 1.44 (s, 2H), 1.34 (d, *J* = 7.0 Hz, 2H); ^13^C NMR (150 MHz, DMSO): *δ* 175.60, 164.02, 140.66, 136.28, 126.43, 125.85, 122.85, 121.04, 118.88, 118.47, 114.88, 112.93, 111.54, 68.55, 42.58, 39.92, 32.14, 28.29, 27.07, 25.29; IR (KBr, cm^−1^): 3416, 3077, 2936, 2854, 1718, 1603, 1512, 1337, 1262, 1179, 1113, 1013, 847, 756; EI-MS m/z: 382.04 (M^+^, 5.38), 130.03(100); Elem. Anal. Calcd. for C_21_H_22_N_2_O_5_: C, 65.96; H, 5.80; N, 7.33. Found: C, 66.23; H, 5.93; N, 7.58.

### 2.13 2-(1*H*-indol-3-yl)-7-(3-(trifluoromethyl)phenoxy)heptanoic acid (10 h)

As a rufous oil in 64% yield. ^1^H NMR (600 MHz, DMSO): *δ* 12.13 (s, 1H), 10.94 (s, 1H), 7.58 (d, *J* = 7.9 Hz, 1H), 7.49 (t, *J* = 7.8 Hz, 1H), 7.34 (d, *J* = 8.0 Hz, 1H), 7.26 (d, *J* = 7.5 Hz, 1H), 7.20 (d, *J* = 11.5 Hz, 3H), 7.06 (t, *J* = 7.4 Hz, 1H), 6.96 (t, *J* = 7.4 Hz, 1H), 4.00 (t, *J* = 6.2 Hz, 2H), 3.72 (d, *J* = 7.5 Hz, 1H), 2.06 (d, *J* = 14.1 Hz, 1H), 1.86–1.75 (m, 1H), 1.73–1.65 (m, 2H), 1.49–1.40 (m, 2H), 1.40–1.28 (m, 2H); ^13^C NMR (150 MHz, CDCl_3_): *δ* 180.64, 159.05, 136.06, 131.66, 131.44, 131.23, 129.83, 126.71, 126.37, 124.90, 123.09, 122.36, 122.09, 121.29, 119.56, 119.08, 117.88, 117.02, 112.95, 111.31, 111.06, 67.93, 42.83, 32.15, 28.75, 27.27, 25.67.; IR (KBr, cm^−1^): 3416, 3069, 2944, 2871, 1710, 1610, 1461, 1328, 1237, 1171, 1120, 890, 789, 748; EI-MS m/z: 405.31 (M^+^, 48.36), 130.04(100); Elem. Anal. Calcd. for C_22_H_22_F_3_NO_3_: C, 65.18; H, 5.47; N, 3.45. Found: C, 65.35; H, 5.70; N, 3.71.

### 2.14 7-(4-Fluoro-3-(trifluoromethyl)phenoxy)-2-(1*H*-indol-3-yl)heptanoic acid (10i)

As a rufous oil in 68% yield. ^1^H NMR (600 MHz, DMSO): *δ* 12.14 (s, 1H), 10.94 (s, 1H), 7.59 (d, *J* = 7.9 Hz, 1H), 7.41 (t, *J* = 9.7 Hz, 1H), 7.35 (d, *J* = 8.1 Hz, 1H), 7.29–7.23 (m, 1H), 7.22 (s, 2H), 7.07 (t, *J* = 7.4 Hz, 1H), 6.97 (t, *J* = 7.4 Hz, 1H), 3.99 (t, *J* = 6.2 Hz, 2H), 3.73 (s, 1H), 2.04 (d, *J* = 13.6 Hz, 1H), 1.86–1.76 (m, 1H), 1.73–1.63 (m, 2H), 1.49–1.41 (m, 2H), 1.39–1.30 (m, 2H); ^13^C NMR (100 MHz, CDCl_3_): *δ* 180.43, 154.73, 154.41, 152.77, 136.08, 128.92, 126.42, 125.15, 123.35, 122.37, 122.04, 121.55, 119.49, 119.20, 119.15, 119.11, 118.49, 118.39, 118.27, 118.18, 117.55, 117.40, 113.17, 112.32, 111.31, 68.54, 43.00, 32.19, 28.74, 27.29, 25.65; IR (KBr, cm^−1^): 3416, 3069, 2945, 2861, 1710, 1618, 1511, 1437, 1320, 1222, 1138, 1046, 814, 748; EI-MS m/z: 423.32 (M^+^, 48.36), 130.05(100); Elem. Anal. Calcd. for C_22_H_21_F_4_NO_3_: C, 62.41; H, 5.00; N, 3.31. Found: C, 62.63; H, 5.24; N, 3.58.

**Synthesis of target compounds 12:** indole (5 mmol, 0.59 g) or 6-bromo-1*H*-indole (5 mmol, 0.98 g), 3, 3-diethoxypropionate (2.5 mmol, 0.48 g), potassium bisulfate (5 mmol, 0.68 g), and 15 ml acetic acid were added successively into a 25-ml round-bottom flask at room temperature. The mixtures were reacted at 80 °C for 5 h. After that, the reaction was quenched with 20 ml water and extracted with ethyl acetate for three times (3 × 50 ml). Then, the typical purification process as aforementioned was followed to produce target compounds **12**.

### 2.15 Ethyl 3,3-di (1*H*-indol-3-yl)propanoate (12a)

As a pale brown solid in 78% yield, m.p. 103.8–104.8 °C. ^1^H NMR (600 MHz, CDCl_3_): *δ* 7.84 (s, 2H), 7.55 (d, *J* = 7.9 Hz, 2H), 7.19 (d, *J* = 8.1 Hz, 2H), 7.10 (t, *J* = 7.5 Hz, 2H), 7.00 (t, *J* = 7.4 Hz, 2H), 6.77 (s, 2H), 5.07 (t, *J* = 7.6 Hz, 1H), 3.99 (q, *J* = 7.0 Hz, 2H), 3.14 (d, *J* = 7.7 Hz, 2H), 1.05 (d, *J* = 7.0 Hz, 3H); ^13^C NMR (150 MHz, CDCl_3_): *δ* 172.76, 136.41, 126.49, 121.74, 119.32, 119.03, 118.35, 111.14, 60.39, 41.15, 30.69, 13.96; IR (KBr, cm^−1^): 3402, 3342, 3061, 2970, 1708, 1461, 1277, 1125, 1096, 1021, 739; EI-MS m/z: 332.07 (M^+^, 22.89), 245.05(100); Elem. Anal. Calcd. for C_21_H_20_N_2_O_2_: C, 75.88; H, 6.06; N, 8.43. Found: C, 75.94; H, 6.31; N, 8.64.

### 2.16 Ethyl 3,3-bis(5-bromo-1*H*-indol-3-yl)propanoate (12b)

As a brown solid in 69% yield, m.p. 161.4–162.2°C. ^1^H NMR (600 MHz, DMSO): *δ* 11.09 (s, 2H), 7.60 (s, 2H), 7.43 (s, 2H), 7.30 (d, *J* = 8.6 Hz, 2H), 7.13 (d, *J* = 8.4 Hz, 2H), 4.88 (t, *J* = 7.7 Hz, 1H), 3.96 (q, *J* = 14.0 Hz, 2H), 3.19 (d, *J* = 7.7 Hz, 2H), 1.05 (t, *J* = 7.0 Hz, 3H); ^13^C NMR (150 MHz, DMSO): *δ* 171.60, 135.17, 127.99, 124.05, 123.34, 121.04, 116.74, 113.47, 110.85, 59.73, 30.26, 13.97; IR (KBr, cm^−1^): 3749, 3424, 3340, 2978, 1718, 1660, 1461, 1286, 1163, 1096, 1038, 872, 789; EI-MS m/z: 490.00 (M^+^, 19.85), 402.95(100); Elem. Anal. Calcd. for C_21_H_18_Br_2_N_2_O_2_: C, 51.45; H, 3.70; N, 5.71. Found: C, 51.71; H, 3.63; N, 5.69.

**Synthesis of target compounds 13:** compound **12a** (2 mmol, 0.66 g) or **12b** (2 mmol, 0.98 g), methyl iodide (6 mmol, 0.85 g) or ethyl iodide (6 mmol, 0.94 g), potassium hydroxide powder (5 mmol, 0.28 g), and 15 ml THF were successively added into a 25-ml round-bottom flask and reacted for 12 h at room temperature. After that, the reaction was quenched with 20 ml water and extracted with ethyl acetate for three times (3 × 50 ml). Then, the typical purification process as aforementioned was followed to obtain the target compounds **13** in 72%–82% yields.

### 2.17 Ethyl 3,3-bis(1-methyl-1*H*-indol-3-yl)propanoate (13a)

As a white solid in 82% yield, m.p. 120.5–121.3°C. ^1^H NMR (600 MHz, CDCl_3_): *δ* 7.59 (d, *J* = 7.9 Hz, 2H), 7.21 (d, *J* = 8.1 Hz, 2H), 7.16 (t, *J* = 7.5 Hz, 2H), 7.02 (t, *J* = 7.4 Hz, 2H), 6.83 (s, 2H), 5.10 (t, *J* = 7.6 Hz, 1H), 4.00 (q, *J* = 7.1 Hz, 2H), 3.61 (s, 6H), 3.15 (d, *J* = 7.7 Hz, 2H), 1.07 (t, *J* = 7.1 Hz, 3H); ^13^C NMR (150 MHz, CDCl_3_): *δ* 172.38, 137.13, 126.94, 126.30, 121.33, 119.50, 118.52, 117.23, 109.05, 60.15, 41.42, 32.50, 30.54, 13.98; IR (KBr, cm^−1^): 3724, 3069, 2979, 2928, 1726, 1618, 1551, 1476, 1371, 1328, 1204, 1151, 1022, 941, 739; EI-MS m/z: 360.05 (M^+^, 11.17), 273.03(100); Elem. Anal. Calcd. for C_23_H_24_N_2_O_2_: C, 76.64; H, 6.71; N, 7.77. Found: C, 76.50; H, 6.41; N, 7.86.

### 2.18 Ethyl 3,3-bis(1-ethyl-1*H*-indol-3-yl)propanoate (13b)

As a pale-yellow solid in 76% yield, m.p. 75.2–76.2°C. ^1^H NMR (600 MHz, CDCl_3_): *δ* 7.58 (d, *J* = 7.9 Hz, 2H), 7.23 (d, *J* = 8.2 Hz, 2H), 7.12 (t, *J* = 7.6 Hz, 2H), 6.99 (t, *J* = 7.5 Hz, 2H), 6.90 (s, 2H), 5.11 (t, *J* = 7.7 Hz, 1H), 4.04–3.93 (m, 6H), 3.17 (d, *J* = 7.7 Hz, 2H), 1.30 (t, *J* = 7.3 Hz, 6H), 1.03 (t, *J* = 7.1 Hz, 3H); ^13^C NMR (150 MHz, CDCl_3_): *δ* 172.38, 136.11, 127.10, 124.58, 121.10, 119.59, 118.38, 117.15, 109.07, 60.04, 41.37, 40.57, 30.79, 15.30, 13.93; IR (KBr, cm^−1^): 3741, 3053, 2969, 2879, 1718, 1543, 1461, 1345, 1253, 1129, 1022, 923, 814, 748; EI-MS m/z: 388.06 (M^+^, 9.81), 301.06(100); Elem. Anal. Calcd. for C_25_H_28_N_2_O_2_: C, 77.29; H, 7.26; N, 7.21. Found: C, 77.11; H, 7.48; N, 7.24.

### 2.19 Ethyl 3,3-bis(5-bromo-1-methyl-1*H*-indol-3-yl)propanoate (13c)

As a white solid in 76% yield, m.p. 159.5–160.3°C. ^1^H NMR (600 MHz, CDCl_3_): *δ* 7.65 (d, *J* = 5.6 Hz, 2H), 7.19 (d, *J* = 8.6 Hz, 2H), 7.04 (d, *J* = 8.7 Hz, 2H), 6.82 (d, *J* = 9.2 Hz, 2H), 4.95 (t, *J* = 7.6 Hz, 1H), 4.03 (q, *J* = 7.1 Hz, 2H), 3.58 (s, 7H), 3.08 (t, *J* = 6.1 Hz, 2H), 1.11 (t, *J* = 7.1 Hz, 3H); ^13^C NMR (150 MHz, CDCl_3_): *δ* 171.90, 135.78, 128.32, 127.39, 124.22, 121.67, 116.34, 112.06, 110.72, 60.33, 51.59, 41.12, 32.68, 30.24, 13.98; IR (KBr, cm^−1^): 3119, 2913, 1728, 1545, 1469, 1427, 1370, 1278, 1204, 1154, 1028, 864, 798; EI-MS m/z: 517.82 (M^+^, 9.80), 430.82(100); Elem. Anal. Calcd. for C_23_H_22_Br_2_N_2_O_2_: C, 53.30; H, 4.28; N, 5.41. Found: C, 53.01; H, 4.35; N, 5.26.

### 2.20 Ethyl 3,3-bis(5-bromo-1-ethyl-1*H*-indol-3-yl)propanoate (13d)

As a white solid in 72% yield, m.p. 148.8–149.6°C. ^1^H NMR (600 MHz, CDCl_3_): *δ* 7.63 (s, 2H), 7.19 (d, *J* = 8.6 Hz, 2H), 7.09 (d, *J* = 8.7 Hz, 2H), 6.94 (s, 2H), 4.96 (t, *J* = 7.7 Hz, 1H), 4.01 (dq, *J* = 21.7, 7.2 Hz, 6H), 3.10 (d, *J* = 7.7 Hz, 2H), 1.34 (t, *J* = 7.3 Hz, 6H), 1.10 (t, *J* = 7.1 Hz, 3H); ^13^C NMR (150 MHz, CDCl_3_): *δ* 171.98, 134.86, 128.56, 125.71, 124.06, 121.92, 116.32, 111.89, 110.74, 60.29, 41.08, 40.89, 30.58, 15.32, 14.01; IR (KBr, cm^−1^): 3741, 3118, 2926, 2878, 1717, 1610, 1545, 1452, 1345, 1272, 1196, 1030, 938, 872, 797; EI-MS m/z: 546.28 (M^+^, 11.58), 459.17(100); Elem. Anal. Calcd. for C_25_H_26_Br_2_N_2_O_2_: C, 54.96; H, 4.80; N, 5.13. Found: C, 54.82; H, 4.63; N, 4.97.

**Synthesis of target compounds 14:** compound **13** (1 mmol), 16 ml mixtures of methanol and water (v/v = 1:1), and sodium hydroxide (5 mmol, 0.2 g) were successively added into a 25-ml round-bottom flask. The mixture was stirred and reacted at refluxing temperature for 2 h. After that, the reaction solution was cooled to room temperature, quenched with 20 ml water, acidified with 2 M HCl to pH = 3, and extracted with ethyl acetate for three times (3 × 25 ml). Then, the typical purification process as aforementioned was performed to obtain the target compounds **14** in 88%–93% yields.

### 2.21 3,3-Di(1*H*-indol-3-yl)propanoic acid (14a)

As a pale-yellow solid in 92% yield, m.p. 176.7–177.6°C. ^1^H NMR (600 MHz, DMSO): *d* 12.11 (s, 1H), 10.83 (s, 2H), 7.53 (d, *J* = 7.9 Hz, 2H), 7.35 (d, *J* = 8.1 Hz, 2H), 7.30 (s, 2H), 7.04 (t, *J* = 7.5 Hz, 2H), 6.91 (t, *J* = 7.4 Hz, 2H), 4.96 (s, 1H), 3.18 (d, *J* = 7.6 Hz, 2H); ^13^C NMR (150 MHz, DMSO): *δ* 173.60, 136.57, 126.45, 122.14, 120.86, 119.03, 118.17, 117.77, 111.48, 40.73, 30.57; IR (KBr, cm^−1^): 3417, 3342, 3059, 2903, 1694, 1618, 1444, 1337, 1287, 1229, 1096, 947, 739; EI-MS m/z: 304.01 (M^+^, 11.27), 117.01(100); Elem. Anal. Calcd. for C_19_H_16_N_2_O_2_: C, 74.98; H, 5.30; N, 9.20. Found: C, 75.25; H, 5.19; N, 9.26.

### 2.22 3,3-Bis(5-bromo-1*H*-indol-3-yl)propanoic acid (14b)

As a rufous solid in 93% yield, m.p. 131.8–132.7°C. ^1^H NMR (600 MHz, DMSO): *δ* 12.11 (s, 1H), 11.08 (s, 2H), 7.59 (s, 2H), 7.41 (s, 2H), 7.30 (d, *J* = 8.5 Hz, 2H), 7.13 (d, *J* = 8.5 Hz, 2H), 4.85 (t, *J* = 7.6 Hz, 1H), 3.11 (d, *J* = 7.6 Hz, 2H); ^13^C NMR (150 MHz, DMSO): *δ* 173.26, 135.22, 128.07, 124.01, 123.35, 121.08, 117.07, 113.51, 110.87, 40.34, 30.22; IR (KBr, cm^−1^): 3749, 3427, 3069, 2961, 1710, 1278, 1212, 1154, 1096, 947, 880, 789; EI-MS m/z: 461.88 (M^+^, 7.52), 194.90(100); Elem. Anal. Calcd. for C_19_H_14_Br_2_N_2_O_2_: C, 49.38; H, 3.05; N, 6.06. Found: C, 49.53; H, 3.15; N, 6.32.

### 2.23 3,3-Bis(1-methyl-1*H*-indol-3-yl)propanoic acid (14c)

As a white solid in 93% yield, m.p. 196.7–197.7°C. ^1^H NMR (600 MHz, DMSO): *δ* 11.25 (s, 1H), 6.67 (d, *J* = 7.9 Hz, 2H), 6.47 (d, *J* = 8.1 Hz, 2H), 6.36 (s, 2H), 6.23 (t, *J* = 7.5 Hz, 2H), 6.08 (t, *J* = 7.4 Hz, 2H), 4.07 (t, *J* = 7.5 Hz, 1H), 2.82 (s, 6H), 2.25 (d, *J* = 7.6 Hz, 2H); ^13^C NMR (150 MHz, DMSO): *δ* 173.37, 136.86, 126.67, 126.49, 121.00, 119.08, 118.32, 117.03, 109.61, 40.83, 32.31, 30.12; IR (KBr, cm^−1^): 3749, 3052, 2920, 1710, 1618, 1545, 1477, 1419, 1328, 1079, 947, 748; EI-MS m/z: 332.17 (M^+^, 18.97), 273.15(100); Elem. Anal. Calcd. for C_21_H_20_N_2_O_2_: C, 75.88; H, 6.06; N, 8.43. Found: C, 75.63; H, 6.12; N, 8.37.

### 2.24 3,3-Bis(1-ethyl-1*H*-indol-3-yl)propanoic acid (14d)

As a white solid in 89% yield, m.p. 185.9–186.7°C. ^1^H NMR (600 MHz, DMSO): *d* 12.05 (s, 1H), 7.49 (d, *J* = 8.0 Hz, 2H), 7.36 (d, *J* = 8.2 Hz, 2H), 7.30 (s, 2H), 7.05 (t, *J* = 7.5 Hz, 2H), 6.90 (t, *J* = 7.5 Hz, 2H), 4.88 (t, *J* = 7.7 Hz, 1H), 4.12 (q, *J* = 7.2 Hz, 4H), 3.09 (d, *J* = 7.7 Hz, 2H), 1.30 (t, *J* = 7.2 Hz, 6H); ^13^C NMR (150 MHz, DMSO): *d* 173.29, 135.83, 126.77, 124.85, 120.82, 119.17, 118.17, 117.01, 109.59, 40.69, 40.08, 30.26, 15.47; IR (KBr, cm^−1^): 3053, 2978, 2879, 2563, 1701, 1610, 1552, 1461, 1346, 1305, 1222, 1146, 947, 748; EI-MS m/z: 360.06 (M^+^, 11.92), 301.06(100); Elem. Anal. Calcd. for C_23_H_24_N_2_O_2_: C, 76.64; H, 6.71; N, 7.77. Found: C, 76.89; H, 6.96; N, 7.64.

### 2.25 3,3-Bis(5-bromo-1-methyl-1*H*-indol-3-yl)propanoic acid (14e)

As a white solid in 88% yield, m.p. 212.8–213.9°C. ^1^H NMR (600 MHz, DMSO): *δ* 12.15 (s, 1H), 7.66 (s, 2H), 7.32 (d, *J* = 8.6 Hz, 4H), 7.20 (d, *J* = 8.6 Hz, 2H), 4.85 (t, *J* = 7.6 Hz, 1H), 3.71 (s, 6H), 3.06 (d, *J* = 7.7 Hz, 2H); ^13^C NMR (150 MHz, DMSO): *δ* 173.08, 135.55, 128.26, 128.23, 123.53, 121.16, 116.44, 111.86, 111.24, 40.66, 32.56, 29.65; IR (KBr, cm^−1^): 3749, 2921, 1710, 1618, 1544, 1477, 1427, 1303, 1146, 1046, 938, 855, 789; EI-MS m/z: 490.01 (M^+^, 8.92), 211.01(100); Elem. Anal. Calcd. for C_21_H_18_Br_2_N_2_O_2_: C, 51.45; H, 3.70; N, 5.71. Found: C, 51.65; H, 3.83; N, 5.93.

### 2.26 3,3-Bis(5-bromo-1-ethyl-1*H*-indol-3-yl)propanoic acid (14f)

As a white solid in 93% yield, m.p. 191.2–192.0°C. ^1^H NMR (600 MHz, DMSO): *δ* 12.12 (s, 1H), 7.57 (s, 2H), 7.45 (s, 2H), 7.38 (d, *J* = 8.7 Hz, 2H), 7.16 (d, *J* = 8.7 Hz, 2H), 4.81 (t, *J* = 7.7 Hz, 1H), 4.14 (q, *J* = 7.1 Hz, 4H), 3.07 (d, *J* = 7.6 Hz, 2H), 1.30 (t, *J* = 7.1 Hz, 6H); ^13^C NMR (150 MHz, DMSO): *δ* 173.09, 134.60, 128.37, 126.75, 123.33, 121.36, 116.33, 111.83, 111.01, 54.95, 40.35, 39.92, 39.78, 39.64, 39.50, 39.36, 39.22, 39.08, 30.02, 15.49; IR (KBr, cm^−1^): 3749, 2978, 2869, 1708, 1610, 1544, 1461, 1295, 1205, 1046, 930, 855, 788; EI-MS m/z: 518.06 (M^+^, 12.42), 459.04(100); Elem. Anal. Calcd. for C_23_H_22_Br_2_N_2_O_2_: C, 53.30; H, 4.28; N, 5.41. Found: C, 53.57; H, 4.48; N, 5.53.

### 2.27 Herbicidal activity test

Herbicidal activities of the synthesized target compounds were evaluated by the typical Petri dish method (Cao et al., 2017). Seeds of barnyard grass (*E. crus-galli*) and rape (*B. napus*) were obtained from the Bioassay Testing and Safety Assessment Center in the Zhejiang Research Institute of Chemical Industry. Each compound (2 mg) was dissolved in 100 μL DMF and emulsified with a drop of Tween-80, and the solution was diluted with water to a concentration of 1000 mg/L and named concentrate solution, parts of which were further diluted to a gradient of 100, 10, and 1 mg/L for use. The solutions of commercial herbicide 2,4-D were also made as the positive control, and water was used as a blank control. The seeds were soaked in warm water (25 °C) for 15 h before use. The test solutions of 9 ml were added to Petri dishes (9 cm diameter) lined with two layers of filter paper on which ten seeds of each of the two model plants were placed. The growth culture was performed in an incubator with a humidity of 75% at 25 °C. The first three days were under complete darkness, followed by the alternating 12/12 diurnal cycle of light (10 Klux) and dark for another five days. Each treatment was repeated three times, and the average inhibition of shoots and roots is calculated according to the following equation:

$$IR=\;\frac{{\bar{l}}_{1}-{\bar{l}}_{2}}{{\bar{l}}_{1}}\;\times 100\%,$$

where ${\bar{l}}_{1}$ is the average length of root/shoot of the blank control treatment and ${\bar{l}}_{2}$ is the average length of root/shoot of the test compound. Data of root and shoot inhibitory rates were subjected to one-way ANOVA, and means were compared by the least significant difference test (*p* < 0.05).

### 2.28 Molecular docking

The crystal structure of TIR1 (PDB code 2P1Q) was downloaded from the Protein Data Bank. The three-dimensional structures of the corresponding active molecule of target compounds **9a**, **9c**, **10d**, **10h**, **14a** and **14d** were generated with the sketch module of SYBYL version 2.0. The docking results were visualized using PyMOL version 1.3 software.

## 3 Results and discussion

For the synthesis of target compounds **9** and **10**, the active hydrogen of NH and COOH groups in indole-3-acetic acid should be protected first by esterification and amidation for ease of its disturbance to the subsequent nucleophilic substitution. For the amidation reaction between indole-3-acetic acid and methyl chloroformate, 30% NaOH aqueous combined with phase transfer catalyst benzyl triethylammonium chloride (TEBAC) is a more suitable base than NaH, resulting in 80% product yield. The base used in the α-substitution reaction of intermediate **3** with alkyl bromide, larger steric-hindrance lithium diisopropylamide (LDA), is superior to *n*-butyllithium because LDA is a poor nucleophilic capacity organic strong base that only interacts with hydrogen proton. Generally, diindolylmethane compounds are synthesized by the reaction of indoles with aldehydes catalyzed by Lewis acid catalyst. In this work, ethyl 3,3-di(1*H*-indol-3-yl)propanoate compounds **12a** and **12b** were efficiently and conveniently synthesized by a “one-pot” reaction between 3, 3-diethoxypropionate and indoles catalyzed by KHSO_4_ and acetic acid.

The herbicidal activities of these target compounds were evaluated by the typical Petri dish method. As shown in Table 1, most of the synthesized target compounds exhibited different levels of inhibition effects on roots and shoots of both dicotyledonous rape (*B. napus*) and monocotyledonous barnyard grass (*E. crus-galli*), and they also had a higher inhibition effect on the root than the shoot for dicotyledonous rape (*B. napus*) while it was reversed for monocotyledonous barnyard grass (*E. cruss-galli*). At 100 mg/L concentration, compounds **10b**–**10d**, **10f**, **10h**, **13b**, **14a**, and **14c** inhibited the root growth of *B. napus* by 93%–97%, and compounds **10d** and **10h** still exhibited 92% and 93% inhibitory rates to the root of *B. napus* at 10 mg/L concentration, respectively. The physiological and biochemical responses of these compounds are in line with the auxinic herbicides, and their inhibitory activities are comparable to positive control 2,4-D. The inhibitory rates to the shoot of barnyard grass (*E. cruss-galli*) range from 60% to 89% while that of rape (*B. napus*) were dramatically decreased to 0%–64% for compounds **9a**–**9c**, **10e**, **10g**-**10i**, **13b**–**13d**, and **14d**–**14e** at 100 mg/L concentration. Compounds **9b**, **10a**, and **13a** inhibited the shoot elongation of barnyard grass (*E. cruss-galli*) by 17%–68% at all test concentrations but completely lost influence on the growth of shoot of rape (*B. napus*), which showed good selectivity to monocotyledonous barnyard grass (*E. cruss-galli*). Compounds **9c**, **10e**, **10g**, **10i**, and **14d** also selectively inhibited the shoot elongation of barnyard grass (*E. cruss-galli*) by 25%–63% at 10 mg/L but 0% to rape (*B. napus*).

**TABLE 1:** Results of Petri dish herbicidal activity test of indole-3-carboxylic acid derivatives.

Compound	Average root inhibitory rate (%)^a^						Average shoot inhibitory rate (%)^a^					
	Rape			Barnyard grass			Rape			Barnyard grass		
	100 mg/L	10 mg/L	1 mg/L	100 mg/L	10 mg/L	1 mg/L	100 mg/L	10 mg/L	1 mg/L	100 mg/L	10 mg/L	1 mg/L
9a	79 ± 2e	55 ± 1 h	37 ± 1i	88 ± 2b	28 ± 1j	24 ± 2i	33 ± 1i	24 ± 2 g	13 ± 1 g	89 ± 2b	42 ± 2i	24 ± 1 g
9b	59 ± 1 h	31 ± 1l	43 ± 1 g	26 ± 2 m	8 ± 1n	4 ± 1o	0 k	0 m	0 k	68 ± 1e	50 ± 1 g	26 ± 2 g
9c	80 ± 1d	66 ± 2f	45 ± 2 g	34 ± 1l	52 ± 1e	13 ± 1l	35 ± 1 h	0 m	0 k	65 ± 1f	63 ± 2d	18 ± 1i
10a	72 ± 2f	64 ± 2f	52 ± 2e	51 ± 1i	33 ± 2i	30 ± 2 g	0 k	0 m	0 k	47 ± 2i	17 ± 1 m	20 ± 1 h
10b	95 ± 1b	69 ± 1e	63 ± 1c	71 ± 2e	41 ± 1 g	17 ± 1 k	58 ± 2d	37 ± 2c	0 k	56 ± 2 h	46 ± 2 h	17 ± 2i
10c	93 ± 2c	73 ± 1d	45 ± 2 g	40 ± 1 k	41 ± 2 g	18 ± 2 k	49 ± 1f	13 ± 1 k	23 ± 1d	41 ± 1j	28 ± 1 k	7 ± 1l
10d	96 ± 1b	92 ± 2b	53 ± 2e	64 ± 1 g	38 ± 1 h	44 ± 1e	63 ± 1c	54 ± 1b	24 ± 2d	32 ± 2l	55 ± 1f	52 ± 1c
10e	73 ± 1f	68 ± 1e	43 ± 1 g	63 ± 2 g	11 ± 1 m	10 ± 1 m	2 ± 1 k	0 m	3 ± 1j	65 ± 1f	25 ± 1l	47 ± 2d
10f	94 ± 1c	78 ± 2c	60 ± 1d	67 ± 1f	37 ± 2 h	43 ± 2e	43 ± 2 g	35 ± 1d	0 k	18 ± 1 m	45 ± 2 h	34 ± 1e
10 g	67 ± 2 g	30 ± 1 m	36 ± 2i	50 ± 1i	37 ± 1 h	24 ± 1i	24 ± 2j	0 m	17 ± 1f	60 ± 1 g	49 ± 1 g	16 ± 1i
10 h	95 ± 2b	93 ± 1b	40 ± 1 h	70 ± 2e	56 ± 1d	3 ± 1o	54 ± 1e	38 ± 2c	2 ± 1j	63 ± 2f	68 ± 2c	0 m
10i	73 ± 1f	68 ± 2e	43 ± 1 g	63 ± 1 g	11 ± 2 m	10 ± 1 m	2 ± 1 k	0 m	0 k	65 ± 1f	25 ± 1l	47 ± 2d
12a	78 ± 2e	65 ± 1f	60 ± 2d	20 ± 2n	35 ± 1i	0p	43 ± 2 g	24 ± 2 g	0 k	66 ± 2e	60 ± 1e	0 m
12b	43 ± 1 k	57 ± 2 h	16 ± 1 k	20 ± 1n	30 ± 1j	6 ± 1n	25 ± 1j	27 ± 1f	0 k	37 ± 1 k	44 ± 2i	10 ± 1 k
13a	60 ± 2 h	46 ± 2j	35 ± 1i	52 ± 2i	24 ± 2 k	20 ± 1j	0 k	0 m	0 k	45 ± 2i	37 ± 1j	18 ± 2i
13b	96 ± 1b	62 ± 1 g	22 ± 2j	34 ± 1l	46 ± 1f	36 ± 1f	64 ± 1c	23 ± 2 h	40 ± 1b	68 ± 1e	37 ± 2j	26 ± 1 g
13c	67 ± 1 g	52 ± 1i	60 ± 2d	62 ± 2 g	28 ± 1j	7 ± 1n	31 ± 2i	15 ± 1j	2 ± 1j	77 ± 2c	3 ± 1 m	10 ± 1 k
13d	78 ± 2e	29 ± 2 m	10 ± 2l	40 ± 1 k	50 ± 2e	34 ± 2f	25 ± 1j	25 ± 2 g	7 ± 2i	63 ± 1f	47 ± 1 h	6 ± 1l
14a	96 ± 1b	36 ± 1 k	42 ± 1 h	35 ± 1l	24 ± 1 k	27 ± 2 h	53 ± 2e	4 ± 1l	0 k	37 ± 2 k	60 ± 2e	30 ± 2f
14b	82 ± 2d	77 ± 1c	60 ± 1d	64 ± 2 g	15 ± 1l	27 ± 1 h	68 ± 1b	31 ± 1e	10 ± 1 h	47 ± 1i	25 ± 2l	0 m
14c	97 ± 1b	48 ± 1j	48 ± 1f	55 ± 1 h	30 ± 2j	21 ± 1j	37 ± 2 h	22 ± 1 h	29 ± 1c	56 ± 2 h	72 ± 1b	64 ± 1a
14d	57 ± 2i	73 ± 1d	76 ± 2b	44 ± 2j	54 ± 1d	11 ± 2l	23 ± 1j	0 m	20 ± 2e	71 ± 1d	50 ± 2 g	15 ± 1j
14e	47 ± 1j	37 ± 2 k	15 ± 1 k	79 ± 1c	87 ± 1b	60 ± 2b	0 k	17 ± 2i	7 ± 1i	79 ± 2c	70 ± 1b	36 ± 2e
14f	55 ± 2i	33 ± 1l	35 ± 2i	75 ± 1d	63 ± 2c	54 ± 1c	48 ± 1f	34 ± 1d	0 k	32 ± 1l	36 ± 2j	22 ± 1 h
2,4-D	100a	100a	100a	100a	100a	75 ± 1a	100a	100a	100a	100a	100a	58 ± 1b
**LSD**_**0.05**_	−3.06	−4.52	−7.57	5.97	−7.06	−6.40	−5.50	−8.52	−10.88	−5.54	−6.98	−3.86

^a^ Each value represents the mean ± SD (*n* = 3), data were processed by SPSS software, and different letters in the same column indicate significant difference among treatments at *p* < 0.05 (LSD test).

Structure–activity relationship analysis revealed that longer alkyl chain of α-alkyl indole-3-acetic acid has no benefit to the inhibitory activity to the root and shoot of barnyard grass (*E. cruss-galli*) (compounds **9a** vs. **9c**) while the alkyl chain length of α-phenyloxyalkyl indole-3-acetic acid has no obvious effect on the inhibitory rates of the both model plants except for compounds **10a** and **10c**. The number and electronic effect of substituents on the phenyl ring exhibited certain influence on the inhibitory rates, which can be verified from the 4-F, 4-NO_2_, or 3-CF_3_ group substituted compounds **10a** and **10c**–**10e**. Comparing the inhibition rates of diindole compounds **12a**–**12b**, **13a**–**13d,** and **14a**–**14f**, it can be seen that the herbicidal activities, especially to dicotyledonous rape (*B. napus*) of a hydrogen atom at fifth position on the indole ring, are generally higher than that of a bromo atom at the fifth position on the indole ring. Furthermore, ethyl group rather than methyl substituted N-H of indole of most of the compounds **12**–**14** led to the increase in inhibitory rates. Whether carboxylic acid or carboxylic ester form is more beneficial to their herbicidal activities is depended on the actual structures of diindoles. For example, the inhibitory rates of diindolepropionic acid compounds **14a**–**14c** are higher than that of diindolepropionic ester compounds **12a**–**12b** and **13a**, whereas, it is reversed for compounds **13b**–**13d** versus **14d**-**14f**.

**FIGURE 5:**
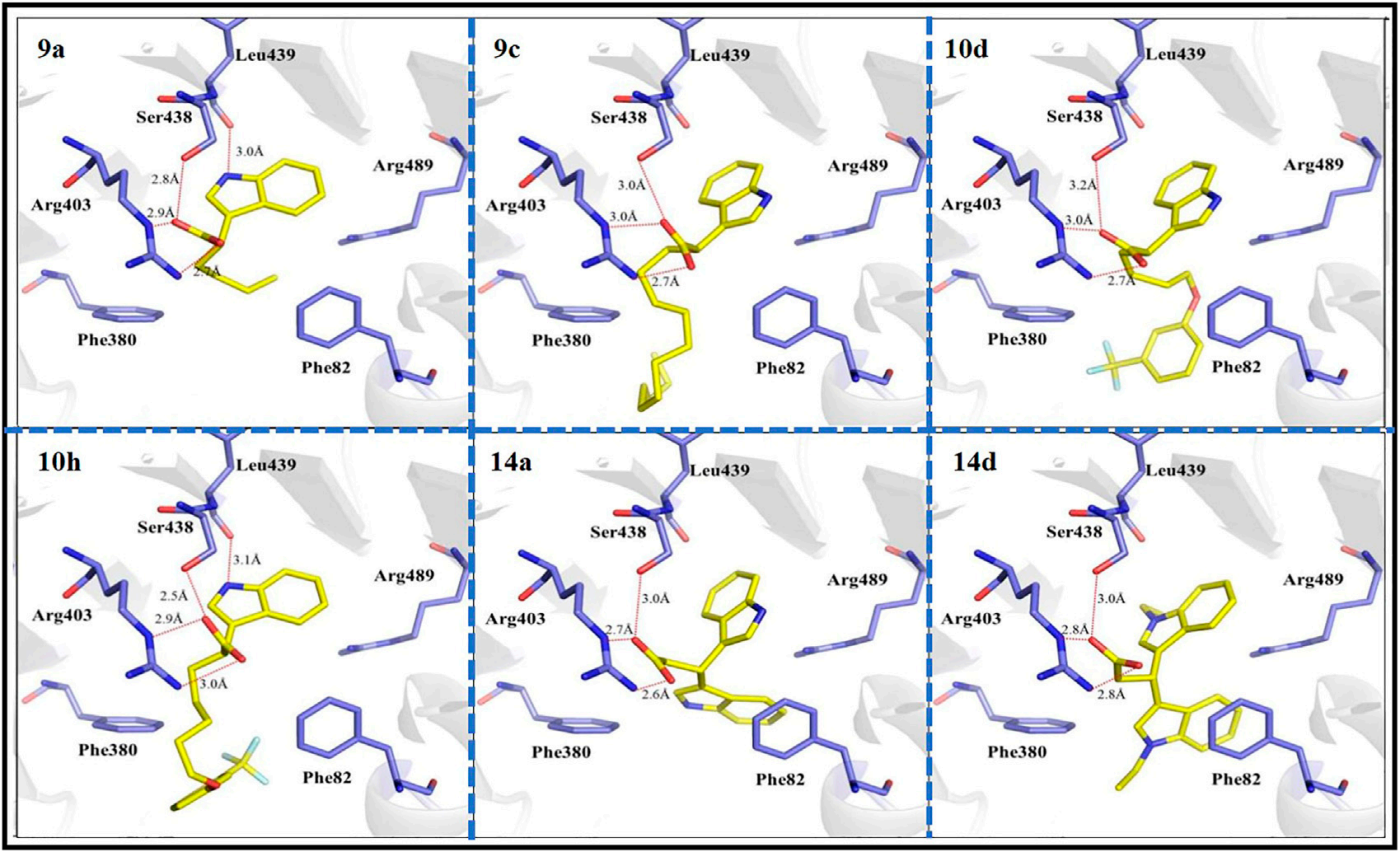
Binding modes of compounds 9a, 9c, 10d, 10h, 14a, and 14d with TIR1 (PDB code 2P1Q) at the active site. The key residues of active sites were represented by blue sticks, and the target compounds are represented by yellow sticks. The oxygen atoms are shown in red, the nitrogen atoms are shown in blue, and the fluorine atoms are shown in light green.

To further investigate the interactions between active small molecules and the target enzyme of herbicides, TIR1 (PDB code 2P1Q) and compounds **9a**, **9c**, **10d**, **10h**, **14a**, and **14d** were selected as target enzymes and ligands, respectively, for molecular docking experiments. As shown in Figure 5, the binding modes of all compounds and TIR1 receptor are basically similar to that of IAA, in which the hydrogen bonds between indole carboxyl group with Arg403 and Ser438 amide groups can be found. Additionally, there is a hydrogen bond interaction between nitrogen atom on indole ring of compounds **9a** and **10h** with the oxygen atom on the main chain of Leu439, which is disappeared when a longer train octyl group or an indole ring replaced the butyl or phenyloxyalkyl group, respectively. This difference in the binding modes is in line with the corresponding herbicidal activity results which are shown in Table 1. The hydrophobic interactions between the terminal long chain of the compounds **10d** and **10h** and Phe380 and Phe82 of TIR1 would contribute significantly to the high affinity of these compounds to TIR1. Furthermore, the tight π–π stacking between the phenyl ring of indole-3-carboxylic acid derivatives and Phe82 of TIR1 demonstrates that the phenyl ring at the end of the α chain facilitates the binding of the compounds to the TIR1 receptor.

## 4 Conclusion

In summary, a series of novel indole-3-carboxylic acid derivatives were rationally designed and synthesized in moderate to excellent yields (54–93%). Petri dish herbicidal activity assay indicates that most of the synthetic compounds showed good to excellent herbicidal activities (60–97% inhibitory rates), especially to dicotyledonous rape (*B. napus*) at 100 mg/L. Compounds **10b**–**10d**, **10f**, **10h**, **13b**, **14a,** and **14c** showed remarkable inhibitory activities in dicotyledonous rape (*B. napus*) with inhibition rates of 93%–97% at 100 mg/L, among which compounds **10d** and **10h** still exhibited 92% and 93% inhibitory rates to the root of *B. napus* at 10 mg/L, respectively. Compounds **9b**, **10a**, and **13a** exhibited good selectivity because they selectively inhibited the elongation of shoot of barnyard grass (*E. cruss-galli*) and are ineffective to that of rape (*B. napus*). Structure–activity relationship analysis indicates that the length of alkyl group and electronic effect have certain influence on the inhibitory activities toward the model plants. Molecular docking results revealed that these synthesized compounds formed tight π–π stacking, hydrogen bond, and hydrophobic interactions with TIR1 protein in the active site. This research provided a supplementary idea and new molecular skeletons for the development of highly effective TIR1 antagonists for herbicide innovation.

## Data Availability

The original contributions presented in the study are included in the article/Supplementary Material; further inquiries can be directed to the corresponding authors.

## References

[B1] Agehara S. Leskovar D. I. (2015). Growth suppression by exogenous abscisic acid and uniconazole for prolonged marketability of bell pepper transplants in commercial conditions. Sci. Hortic. 194, 118–125. 10.1016/j.scienta.2015.08.010

[B2] Bandurski R. S. Cohen J. D. Slovin J. P. Reinecke D. M. (1995). Auxin biosynthesis and metabolism. Plant hormones. Springer Netherlands, p39–65.

[B3] Bauer A. Luetjohann J. Rohn S. Kuballa J. Jantzen E. (2018). Development of an LC-MS/MS method for simultaneous determination of the quaternary ammonium herbicides paraquat, diquat, chlormequat, and mepiquat in plant-derived commodities. Food Anal. Methods 11, 2237–2243. 10.1007/s12161-018-1201-6

[B4] Bell J. L. Schmitzer P. R. Walsh T. A. (2019). New auxin mimics and herbicides. Wiley-VCH Verlag GmbH & Co. KGaA.

[B5] Cao Y.-Y. Mao D.-J. Wang W.-W. Du X.-H. (2017). Kresoxim-methyl derivatives: Synthesis and herbicidal activities of (Pyridinylphenoxymethylene)phenyl methoxyiminoacetates. J. Agric. Food Chem. 65 (30), 6114–6121. 10.1021/acs.jafc.7b02710 28683548

[B6] Dharmasiri N. Dharmasiri S. Estelle M. (2005). The F-box protein TIR1 is an auxin receptor. Nature 435, 441–445. 10.1038/nature03543 15917797

[B7] Du F.-Y. Li X.-M. Song J.-Y. Li C.-S. Wang B.-G. (2014). Anthraquinone derivatives and an orsellinic acid ester from the marine alga-derived endophytic fungus *Eurotium cristatum* EN-220. Helv. Chim. Acta 97 (7), 973–978. 10.1002/hlca.201300358

[B8] Duca D. R. Glick B. R. (2020). Indole-3-acetic acid biosynthesis and its regulation in plant-associated bacteria. Appl. Microbiol. Biotechnol. 104, 8607–8619. 10.1007/s00253-020-10869-5 32875364

[B9] Ebrahimzadeh H. Shariatpanahi M. E. Ahmadi B. Soltanloo H. Lotf M. Zarif E. (2018). Efficient parthenogenesis induction and *in vitro* haploid plant regeneration in cucumber (cucumis sativus L.) using putrescine, spermidine, and cycocel. J. Plant Growth Regul. 37, 1127–1134. 10.1007/s00344-018-9803-1

[B10] Enders T. A. Strader L. C. (2015). Auxin activity: Past, present, and future. Am. J. Bot. 102 (2), 180–196. 10.3732/ajb.1400285 25667071 PMC4854432

[B11] Gianessi L. P. (2013). The increasing importance of herbicides in worldwide crop production. Pest Manag. Sci. 69 (10), 1099–1105. 10.1002/ps.3598 23794176

[B12] Grossmann K. (2010). Auxin herbicides: Current status of mechanism and mode of action. Pest Manag. Sci. 66 (2), 113–120. 10.1002/ps.1860 19823992

[B13] Grossmann K. (2003). Mediation of herbicide effects by hormone interactions. J. Plant Growth Regul. 22, 109–122. 10.1007/s00344-003-0020-0

[B14] Hayashi K.-I. Neve J. Hirose M. Kuboki A. Shimada Y. Kepinski S. (2012). Rational design of an auxin antagonist of the SCFTIR1 auxin receptor complex. ACS Chem. Biol. 7 (3), 590–598. 10.1021/cb200404c 22234040

[B15] Hayashi K.-I. Tan X. Zheng N. Hatate T. Kimura Y. Kepinski S. (2008). Small-molecule agonists and antagonists of F-box protein-substrate interactions in auxin perception and signaling. Proc. Natl. Acad. Sci. U. S. A. 105, 5632–5637. 10.1073/pnas.0711146105 18391211 PMC2291130

[B16] Hayashi K.-I. (2012). The interaction and integration of auxin signaling components. Plant Cell Physiol. 53 (3), 965–975. 10.1093/pcp/pcs035 22433459

[B17] Heap I. (2022). The international survey of herbicide resistant weeds. Availableat: http://www.weedscience.org (Accessed June 18, 2022).

[B18] Indegit K. Duke S. O. (2003). Ecophysiological aspects of allelopathy. Planta 217, 529–539. 10.1007/s00425-003-1054-z 12811559

[B19] Jeschke P. Witschel M. Wolfgang K. Schirmer U. (2019). New auxin mimics and herbicides. Wiley-VCH Verlag GmbH & Co. KGaA, p303–350.

[B20] Kepinski S. Leyser O. (2005). The *Arabidopsis* F-box protein TIR1 protein is an auxin receptor. Nature 435, 446–451. 10.1038/nature03542 15917798

[B21] Korasick D. A. Jez J. M. Strader L. C. (2015). Refining the nuclear auxin response pathway through structural biology. Curr. Opin. Plant Biol. 27, 22–28. 10.1016/j.pbi.2015.05.007 26048079 PMC4618177

[B22] Leyser O. (2002). Molecular genetics of auxin signaling. Annu. Rev. Plant Biol. 53, 377–398. 10.1146/annurev.arplant.53.100301.135227 12221981

[B23] Marumo S. Hattori H. Abe H. (1971). Chromatography of a new natural auxin, 4-chloroindolyl-3-acetic acid and related chloro derivatives. Anal. Biochem. 40, 488–490. 10.1016/0003-2697(71)90411-8 5551560

[B24] Mithila J. Christopher Hall J. Johnson W. G. Kelley K. B. Riechers D. E. (2011). Evolution of resistance to auxinic herbicides: Historical perspectives, mechanisms of resistance, and implications for broadleaf weed management in agronomic crops. Weed Sci. 59, 445–457. 10.1614/WS-D-11-00062.1

[B25] Moss S. (2019). Integrated weed management (IWM): Why are farmers reluctant to adopt non-chemical alternatives to herbicides? Pest Manag. Sci. 75 (5), 1205–1211. 10.1002/ps.5267 30450751

[B26] Myo E. M. Ge B.-B. Ma J.-J. Cui H.-L. Liu B.-H. Shi L.-M. (2019). Indole-3-acetic acid production by Streptomyces fradiae NKZ-259 and its formulation to enhance plant growth. BMC Microbiol. 19, 155. 10.1186/s12866-019-1528-1 31286877 PMC6615096

[B27] Pieterse C. M. J. Van der Does D. Zamioudis C. Leon-Reyes A. Van Wees S. C. M. (2012). Hormonal modulation of plant immunity. Annu. Rev. Cell Dev. Biol. 28 (1), 489–521. 10.1146/annurev-cellbio-092910-154055 22559264

[B28] Rauzan B. M. Lorsbach B. A. (2021). Designing sustainable crop protection actives. Washington, DC: American Chemical Society.

[B29] Ross J. J. O’Neill D. P. Wolbang C. M. Symons G. M. Reid J. B. (2001). Auxin-gibberellin interactions and their role in plant growth. J. Plant Growth Regul. 20, 346–353. 10.1007/s003440010034 11986760

[B30] Salehin M. Bagchi R. Estelle M. (2015). SCFTIR1/AFB-based auxin perception: Mechanism and role in plant growth and development. Plant Cell 27 (1), 9–19. 10.1105/tpc.114.133744 25604443 PMC4330579

[B31] Sustainability (2021). Corteva agriscience. Availableat: https://www.corteva.com/sustainability.html (Accessed July 07, 2021).

[B32] Tan W.-M. Hou N. Pang S. Zhu X. F. Li Z. H. Wen L. X. (2012). Improved biological effects of uniconazole using porous hollow silica nanoparticles as carriers. Pest Manag. Sci. 68 (3), 437–443. 10.1002/ps.2288 21997963

[B33] Tudi M. Daniel Ruan H. Wang L. Lyu J. Sadler R. Connell D. (2021). Agriculture development, pesticide application and its impact on the environment. Int. J. Environ. Res. Public Health 18 (3), 1112. 10.3390/ijerph18031112 33513796 PMC7908628

[B34] Tung S. A. Huang Y. Hafeez A. Ali S. Khan Z. Souliyanonh B. (2018). Mepiquat chloride effects on cotton yield and biomass accumulation under late sowing and high density. Field Crops Res. 215, 59–65. 10.1016/j.fcr.2017.09.032

[B35] Verma V. C. Kharwar R. N. Strobel G. A. (2009). Chemical and functional diversity of natural products from plant associated endophytic fungi. Nat. Prod. Commun. 4 (11), 1934578X0900401. 10.1177/1934578X0900401114 19967984

[B36] Zhao Y. D. (2010). Auxin biosynthesis and its role in plant development. Annu. Rev. Plant Biol. 61, 49–64. 10.1146/annurev-arplant-042809-112308 20192736 PMC3070418

