# Supplementary material for: Design, synthesis, and herbicidal activity of indole-3-carboxylic acid derivatives as potential transport inhibitor response 1 antagonists
Source: Front Chem. 2022 Jul 26;10:975267. doi: 10.3389/fchem.2022.975267 (PMC9360422; doi:10.3389/fchem.2022.975267)
Supplement: Supplementary file 1 [file DataSheet1.pdf]

Supplementary Material

Contents

Copes of ^{1}H NMR and ^{13}C NMR spectra of the synthetic compounds **9a~9c**, **10a~10i**, **12a~12b**, **13a~13d**, and **14a~14f**

1 Supplementary Tables and Figures

1.1 Supplementary Figures

Copes of ^{1}H NMR and ^{13}C NMR spectra of the synthetic compounds 9a~9c, 10a~10i, 12a~12b, 13a~13d, and 14a~14f.

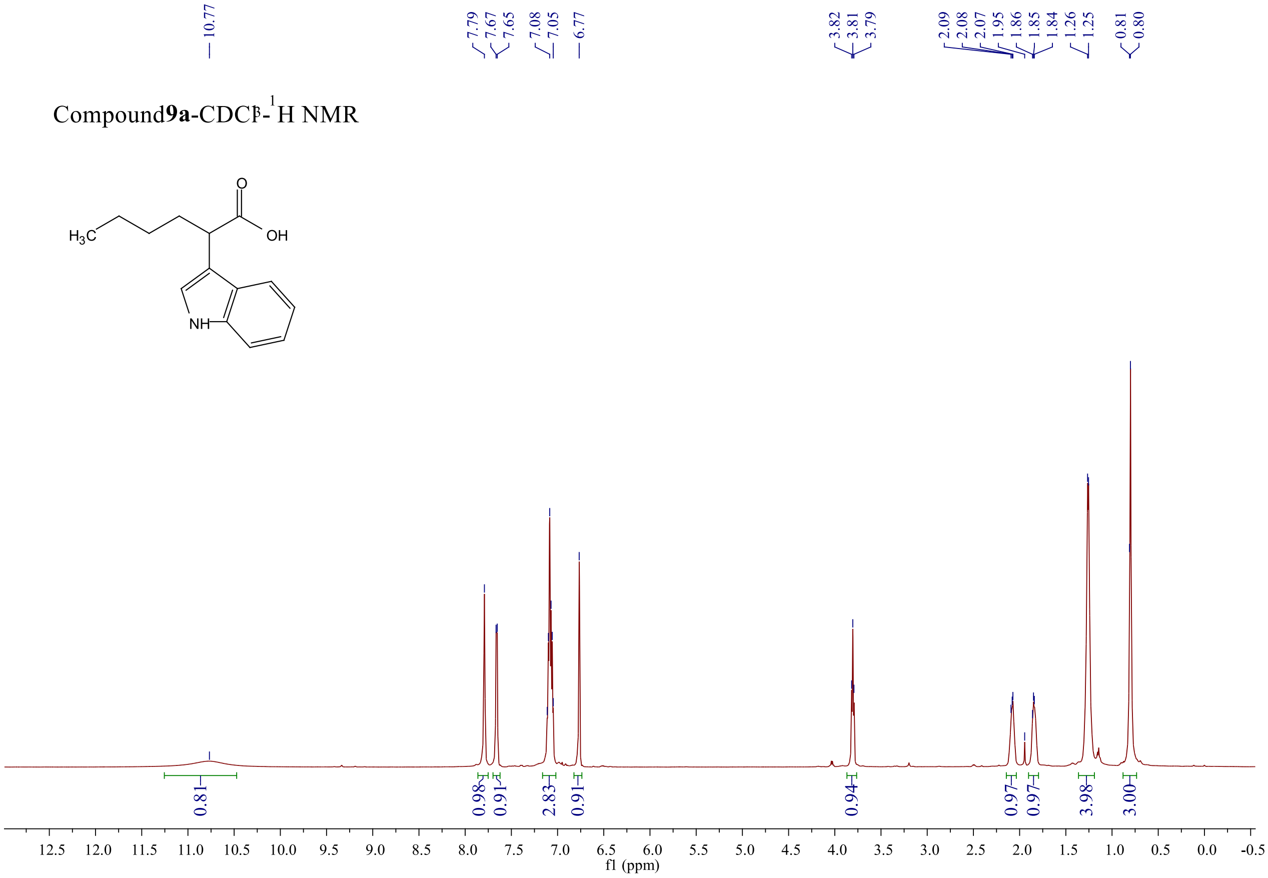

^{1}H NMR spectrum of compound **9a**

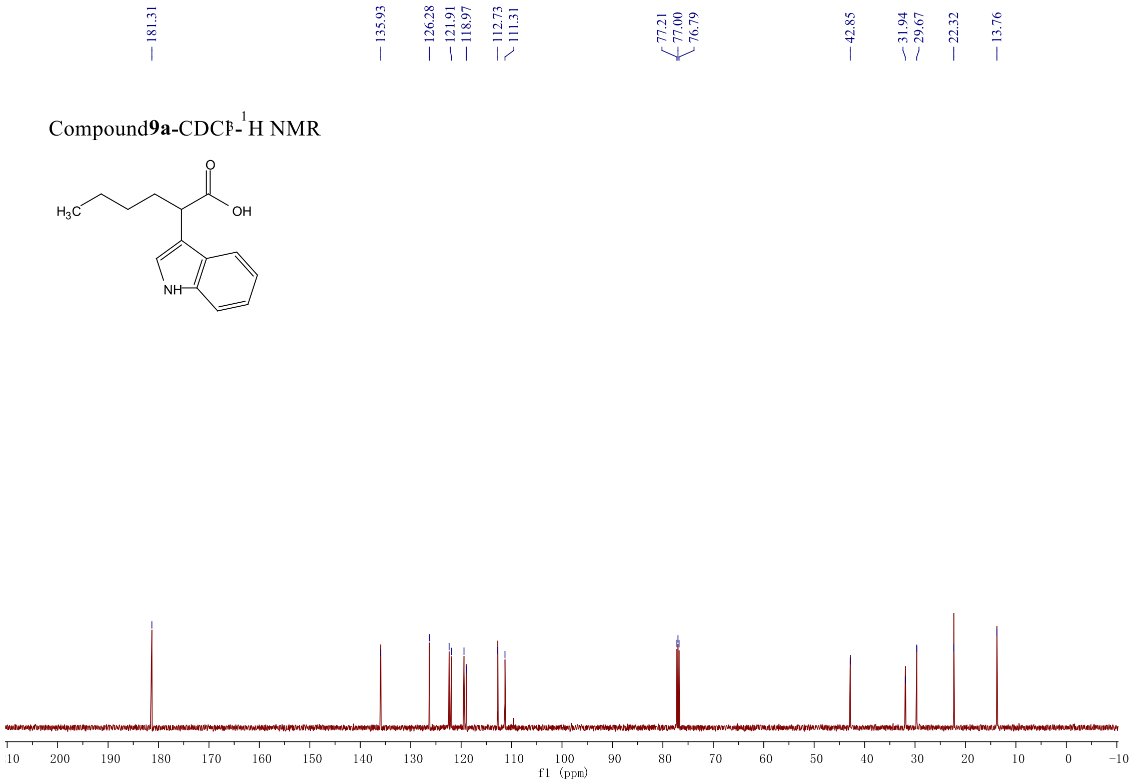

^{13}C NMR spectrum of compound **9a**

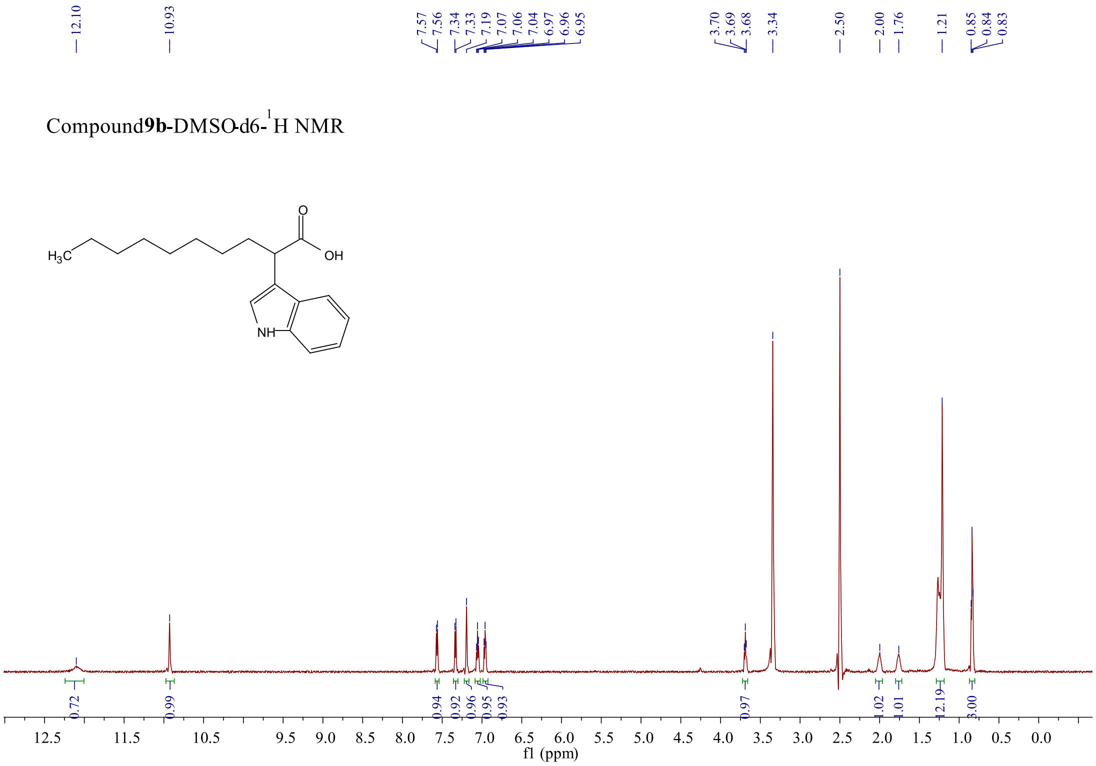

^{1}H NMR spectrum of compound **9b**

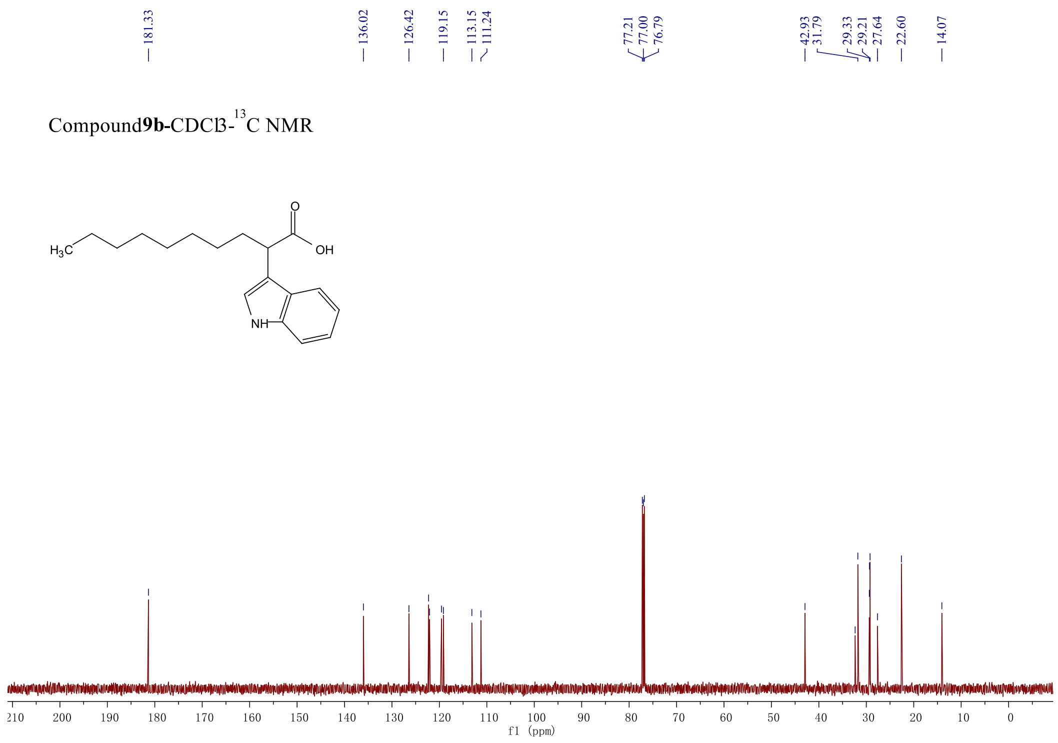

^{13}C NMR spectrum of compound **9b**

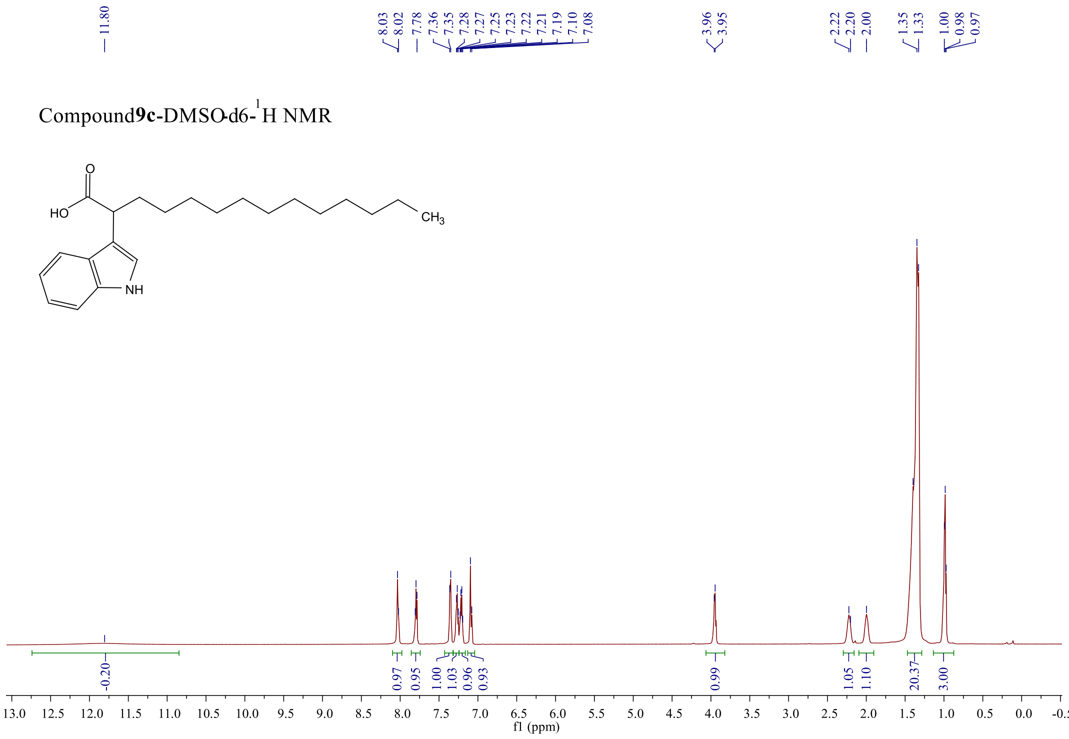

^{1}H NMR spectrum of compound **9c**

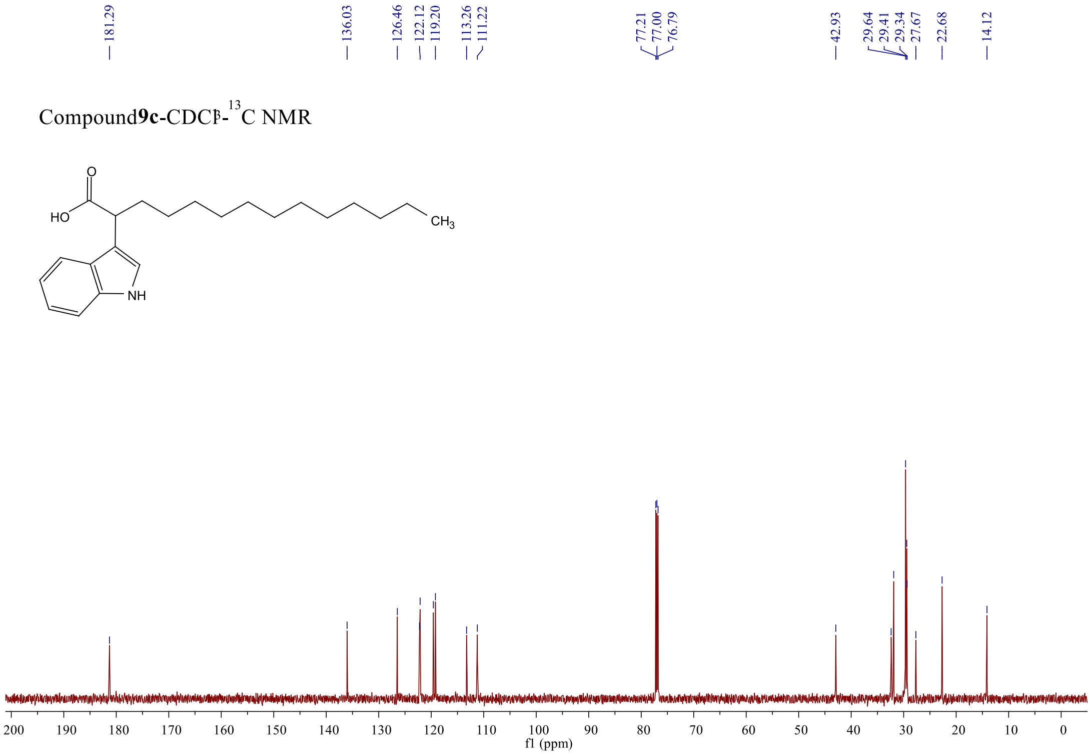

^{13}C NMR spectrum of compound **9c**

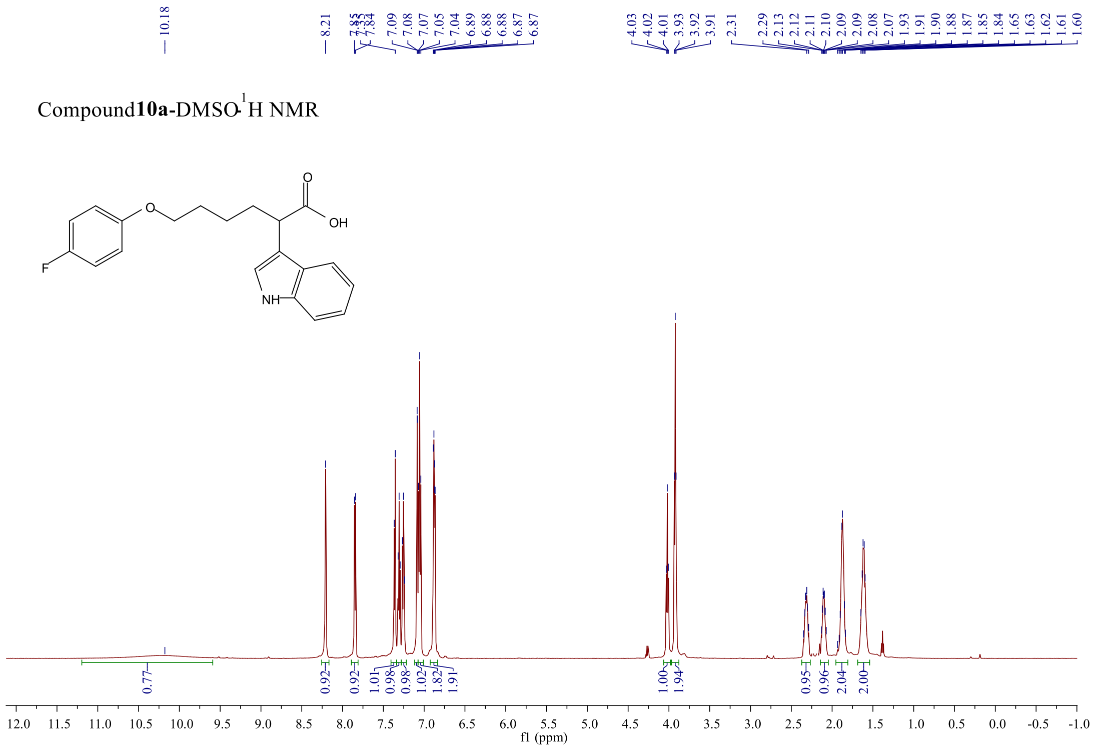

^{1}H NMR spectrum of compound **10a**

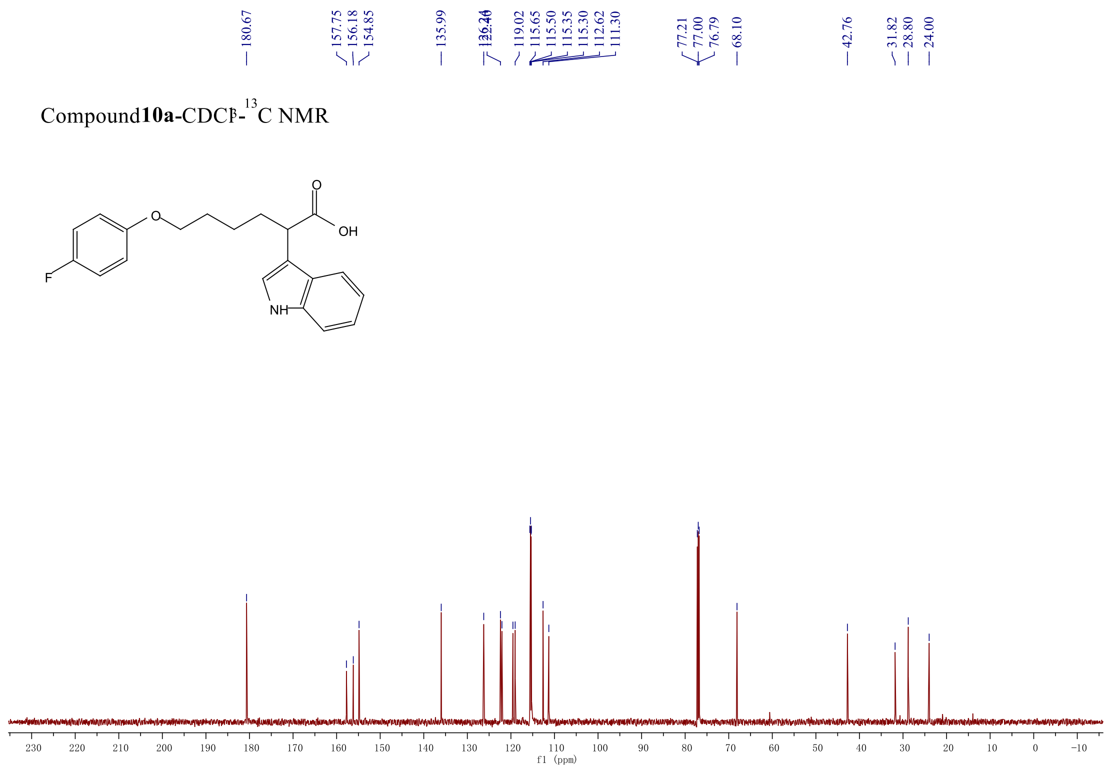

^{13}C NMR spectrum of compound **10a**

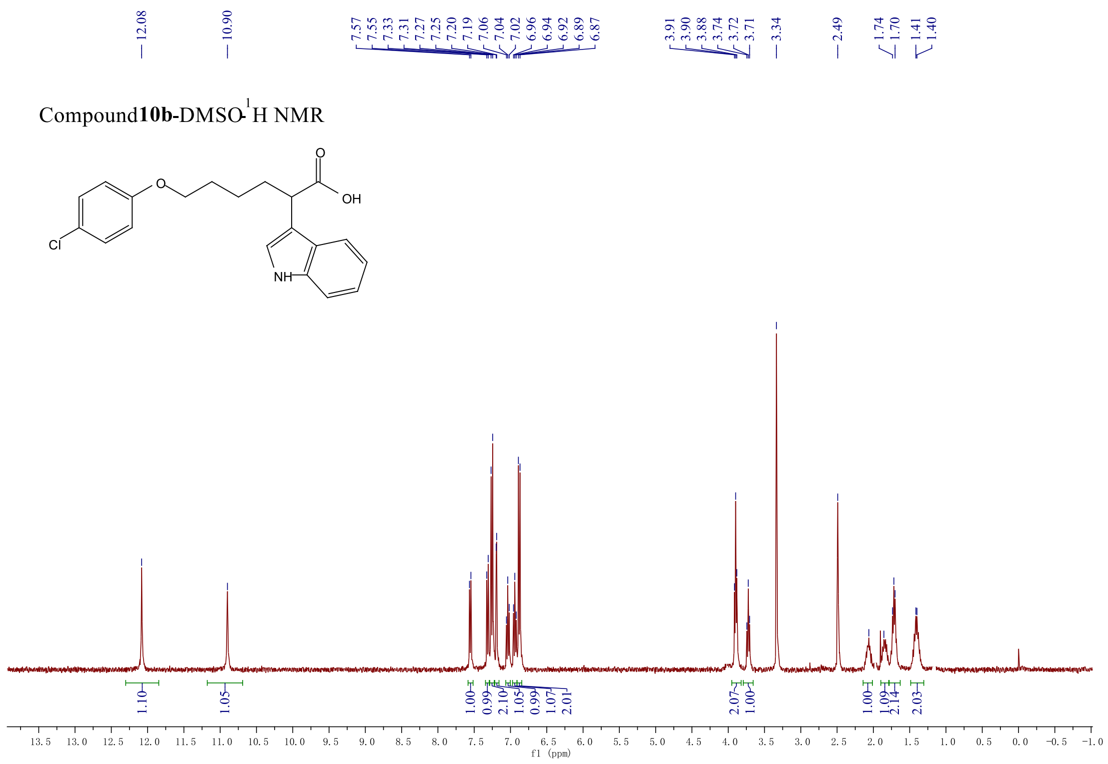

^{1}H NMR spectrum of compound **10b**

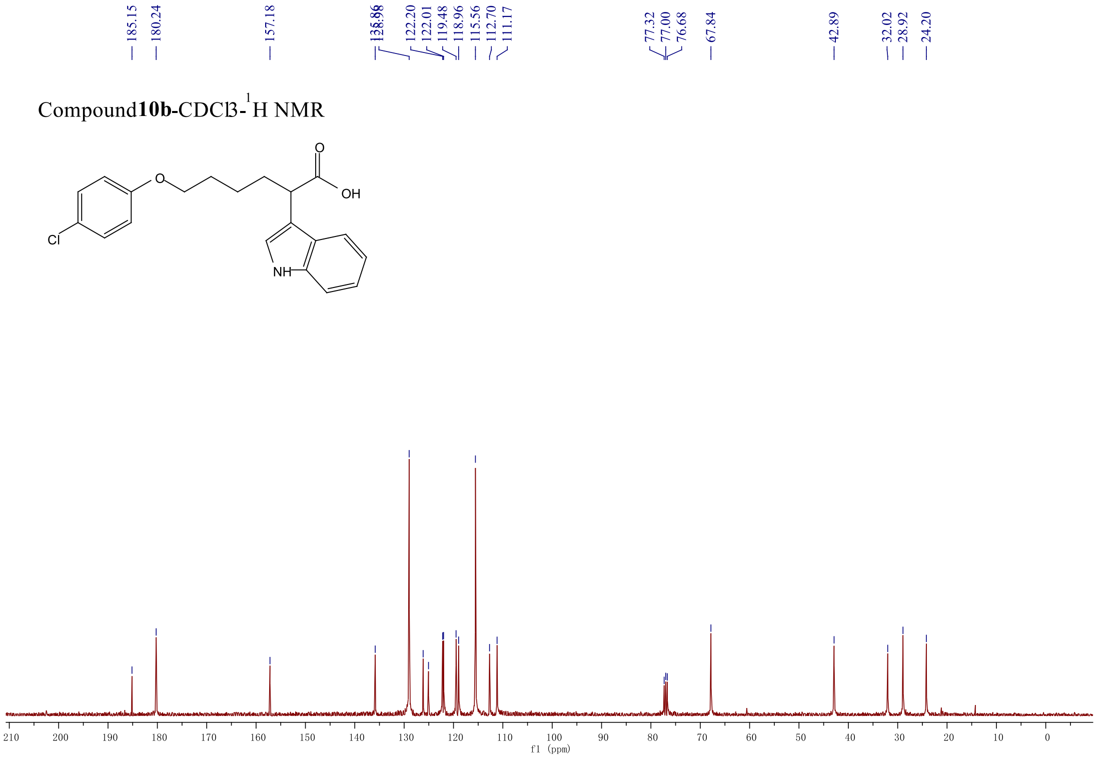

^{13}C NMR spectrum of compound **10b**

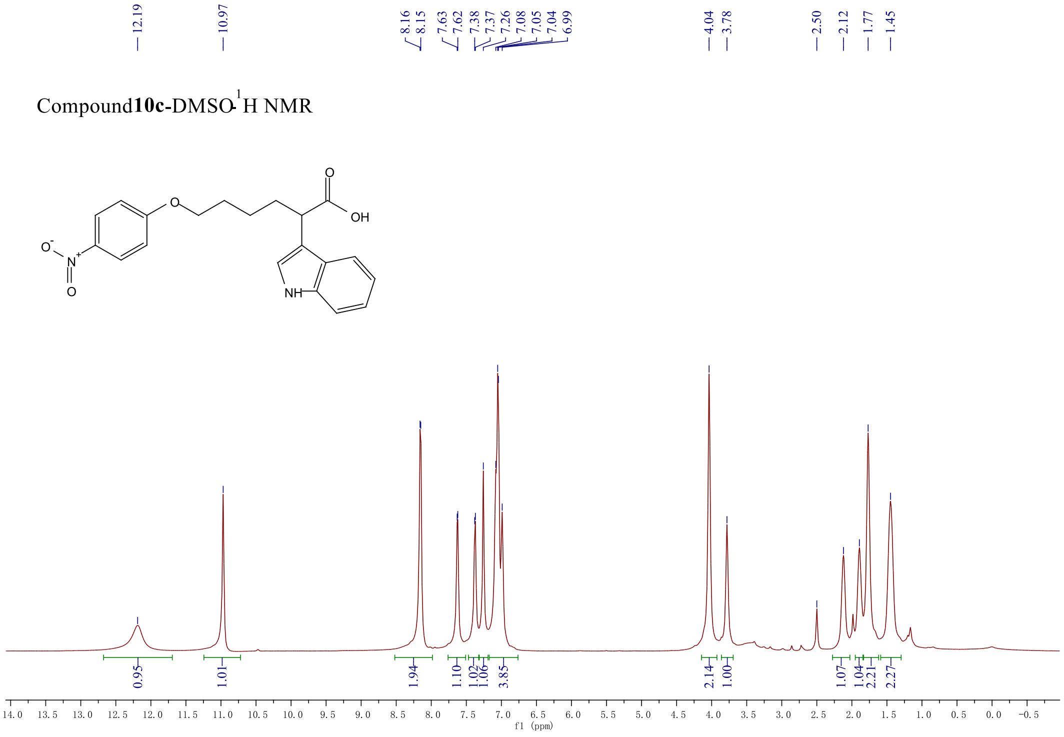

^{1}H NMR spectrum of compound **10c**

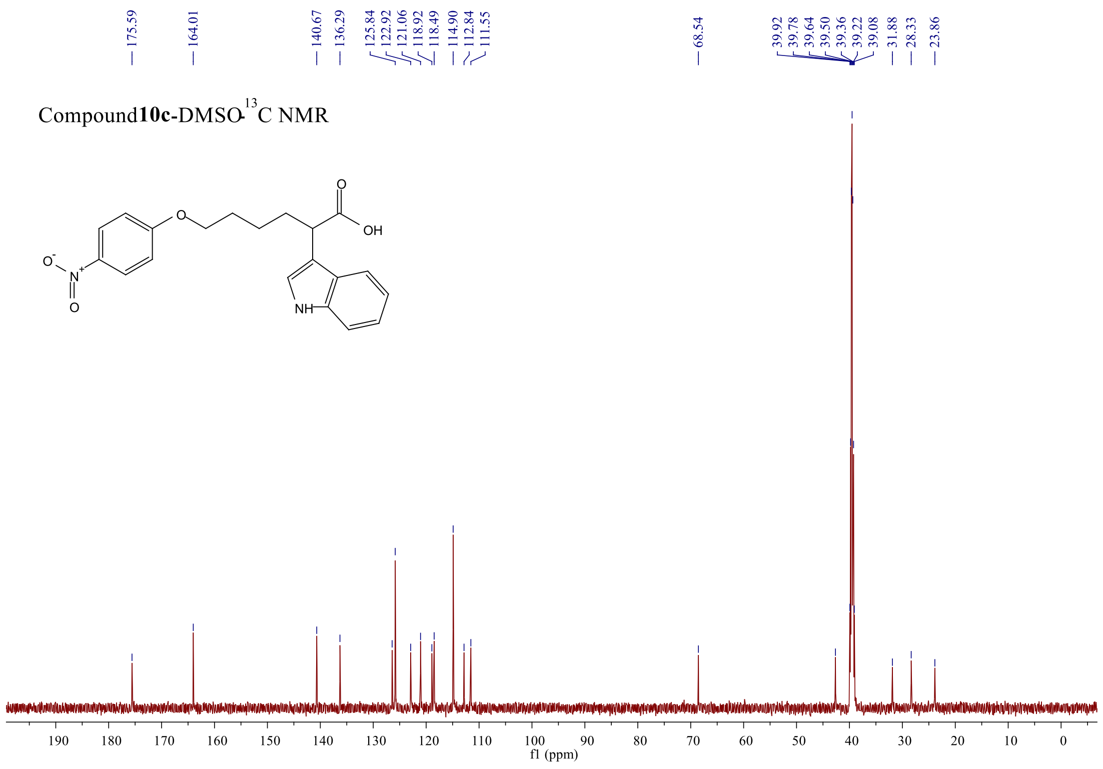

^{13}C NMR spectrum of compound **10c**

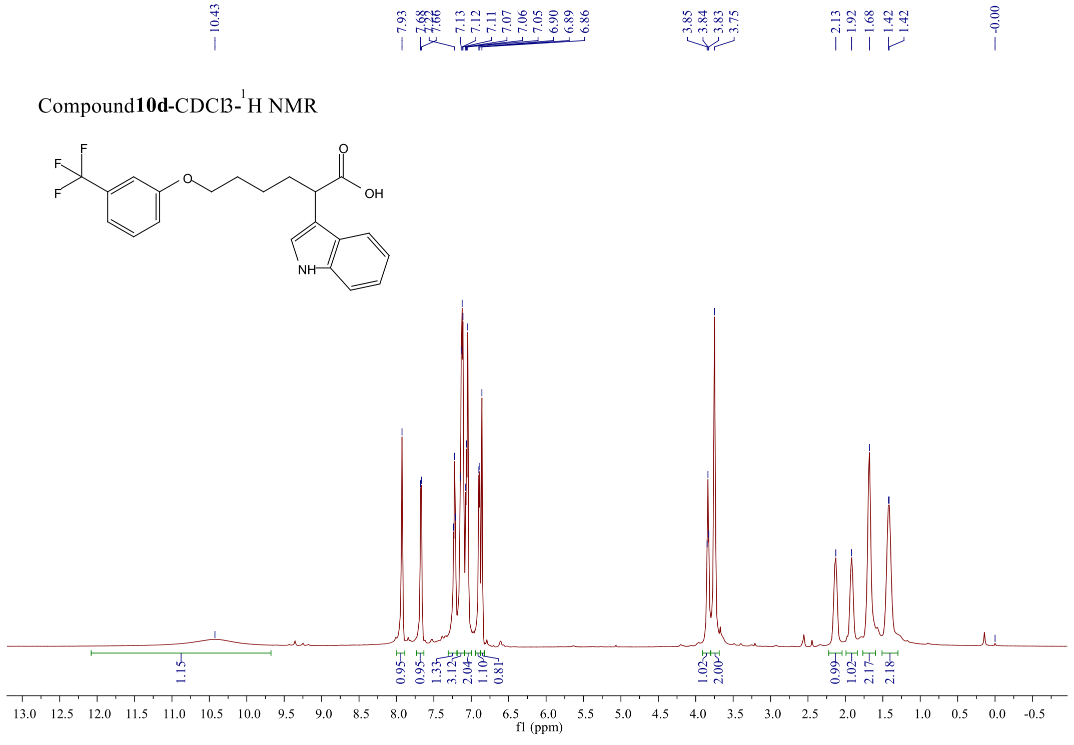

^{1}H NMR spectrum of compound **10d**

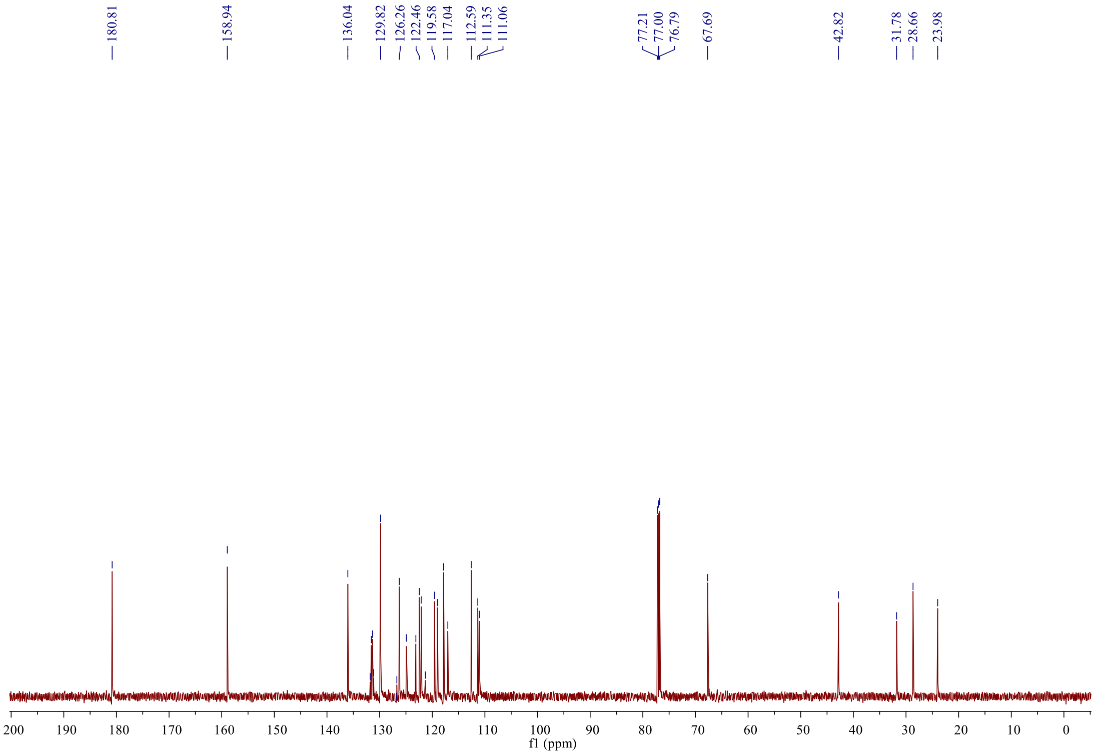

^{13}C NMR spectrum of compound **10d**

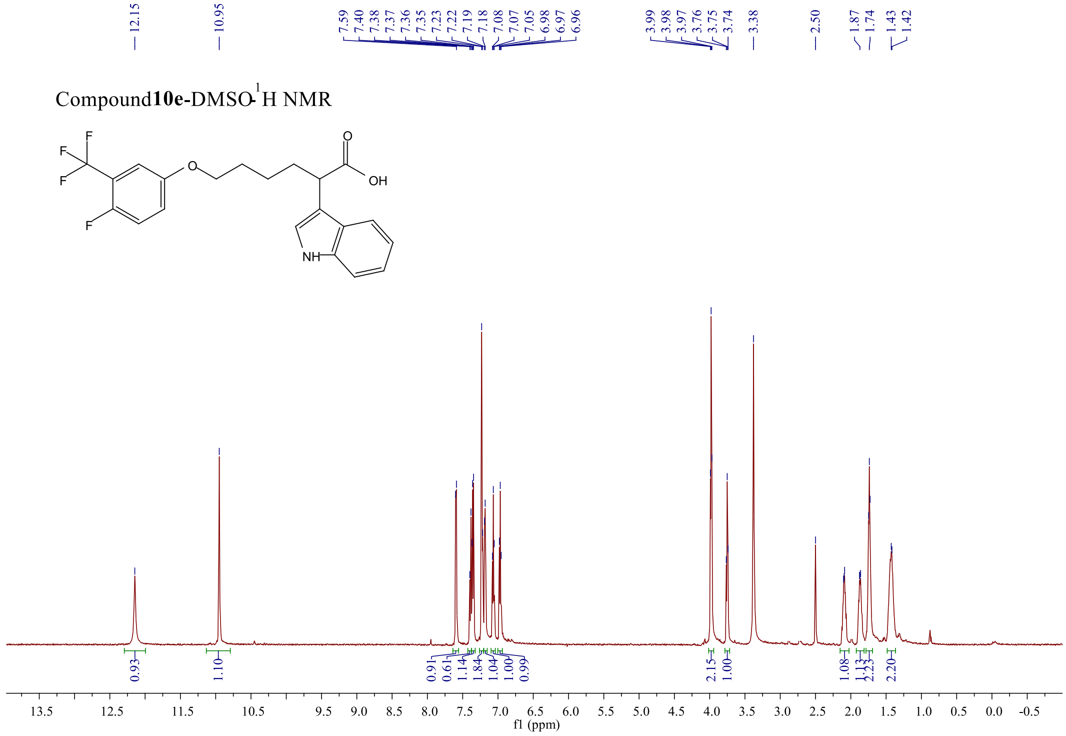

^{1}H NMR spectrum of compound **10e**

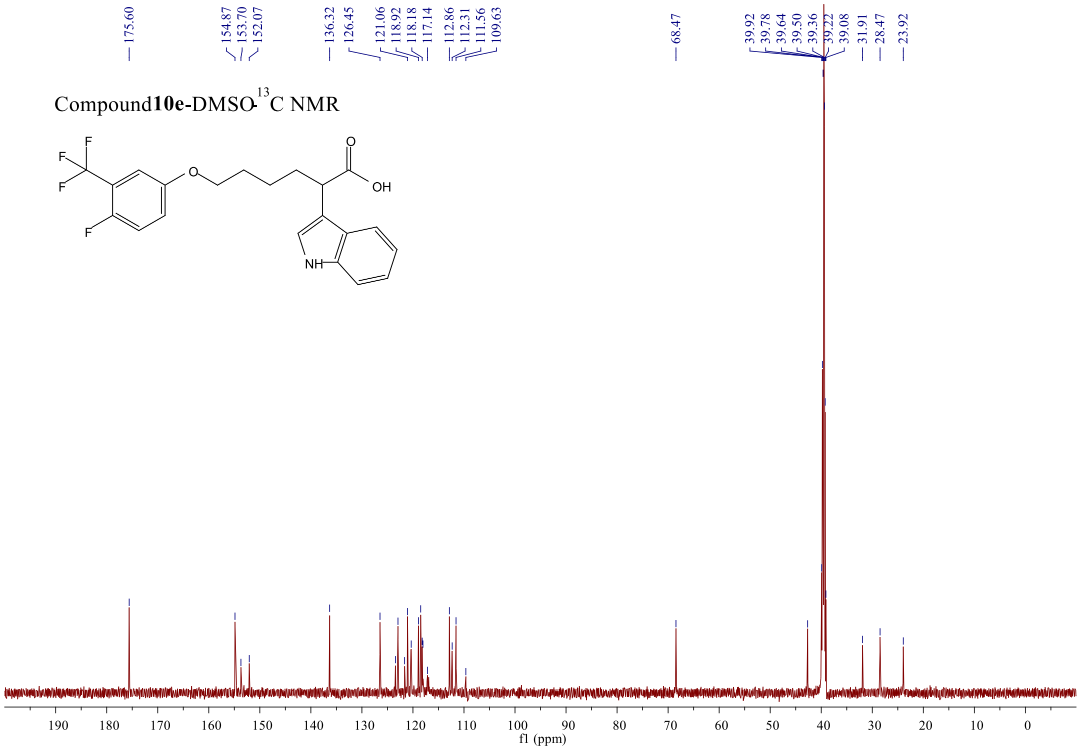

^{13}C NMR spectrum of compound **10e**

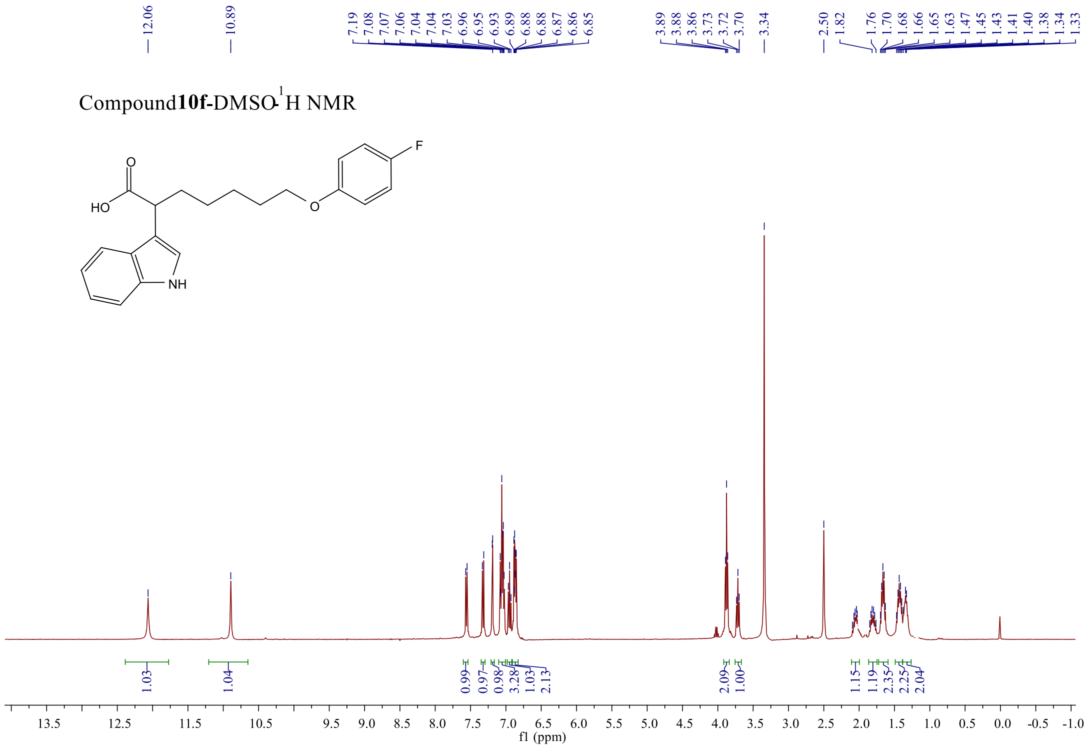

^{1}H NMR spectrum of compound **10f**

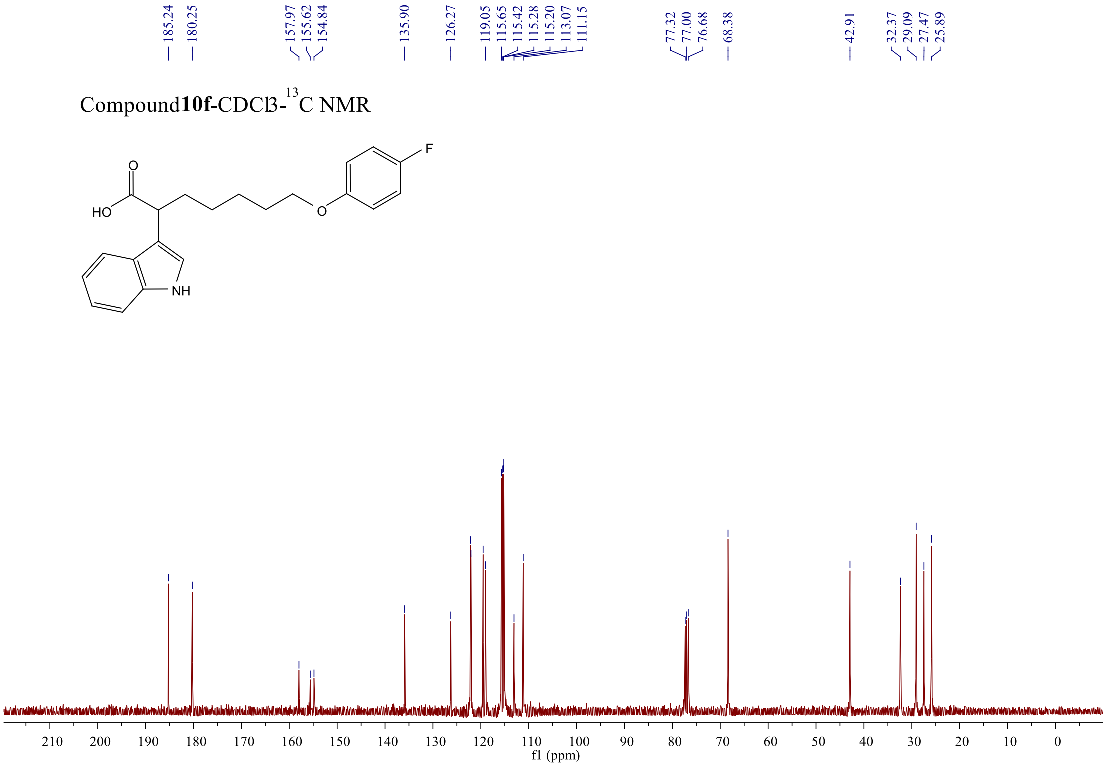

^{13}C NMR spectrum of compound **10f**

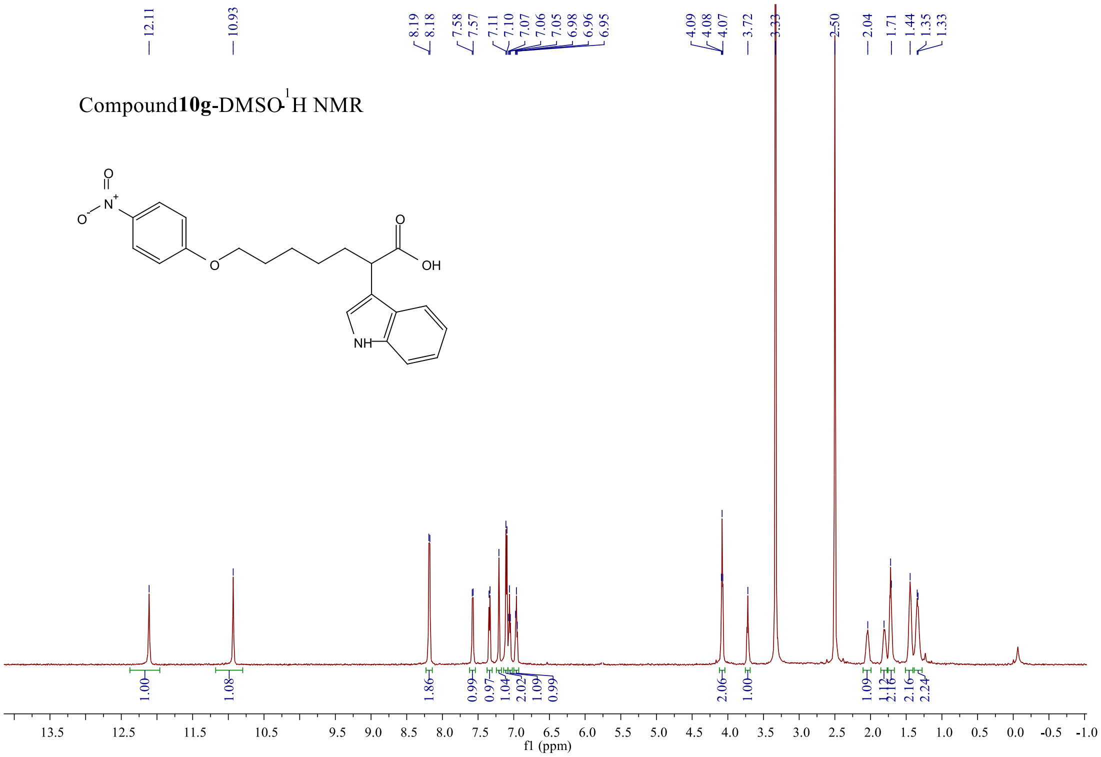

^{1}H NMR spectrum of compound **10g**

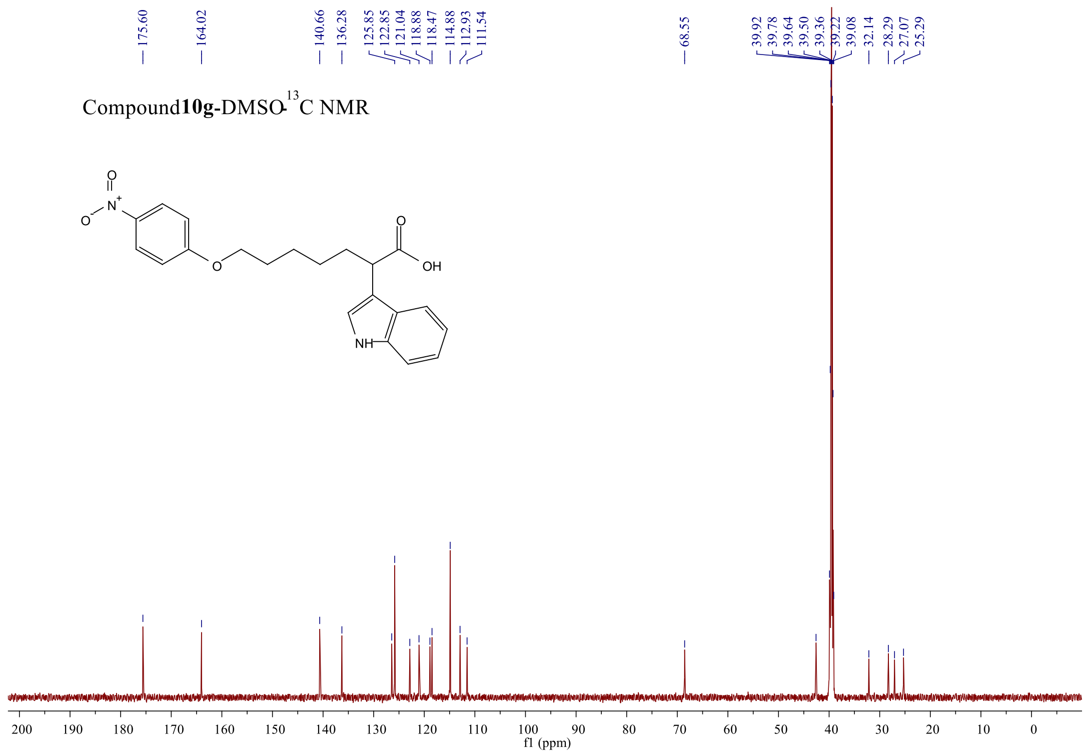

^{13}C NMR spectrum of compound **10g**

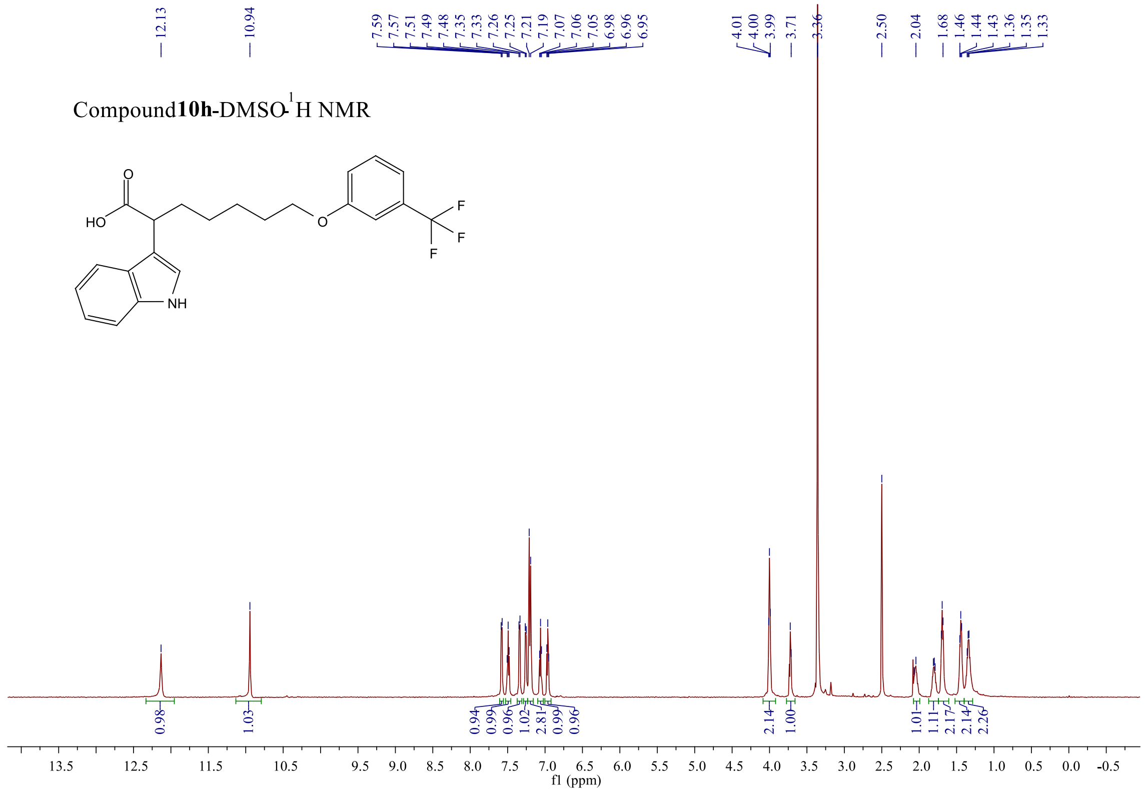

^{1}H NMR spectrum of compound **10h**

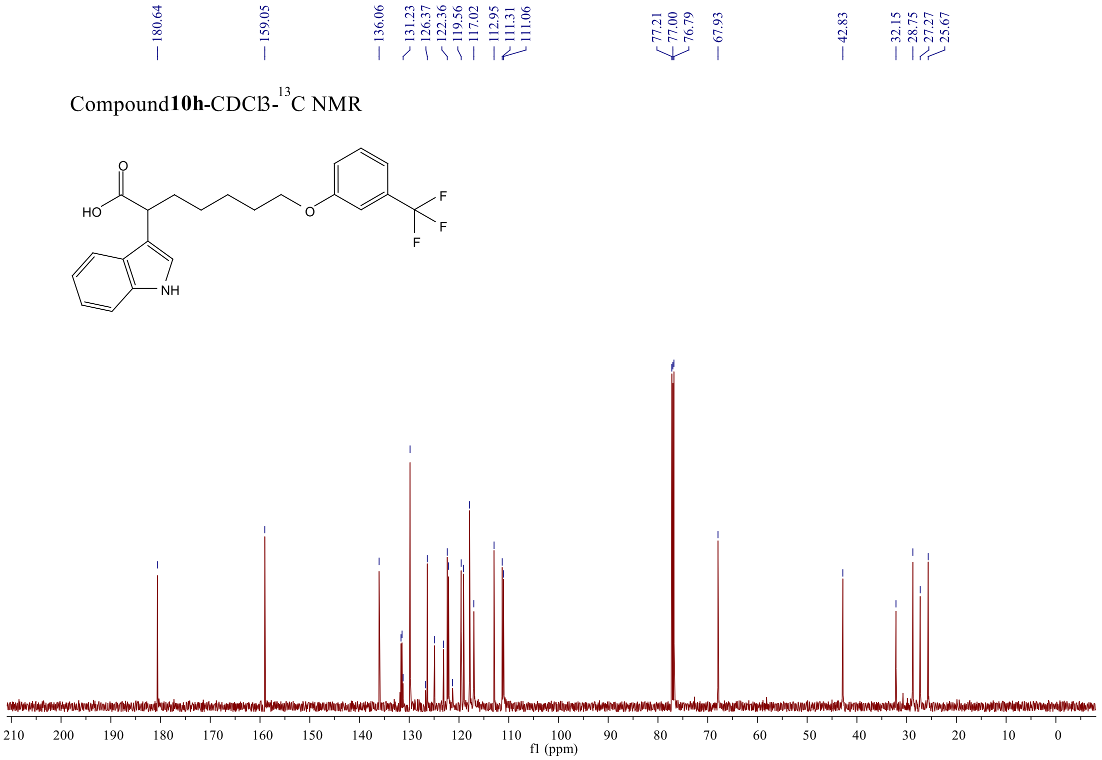

^{13}C NMR spectrum of compound **10h**

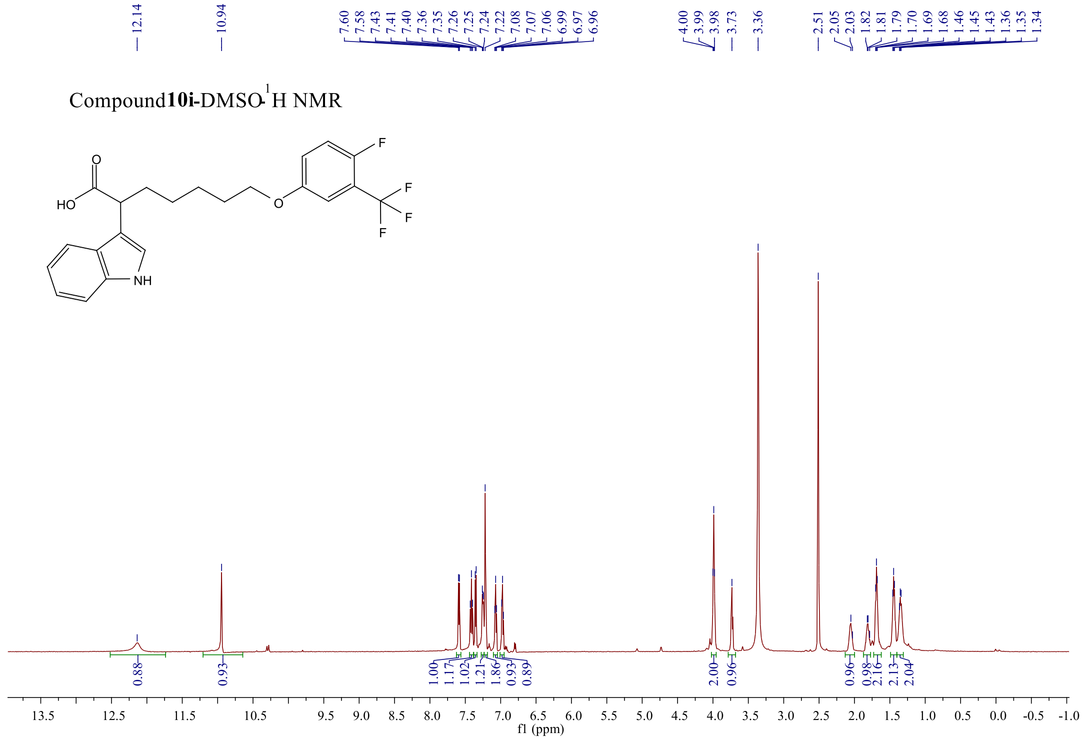

^{1}H NMR spectrum of compound **10i**

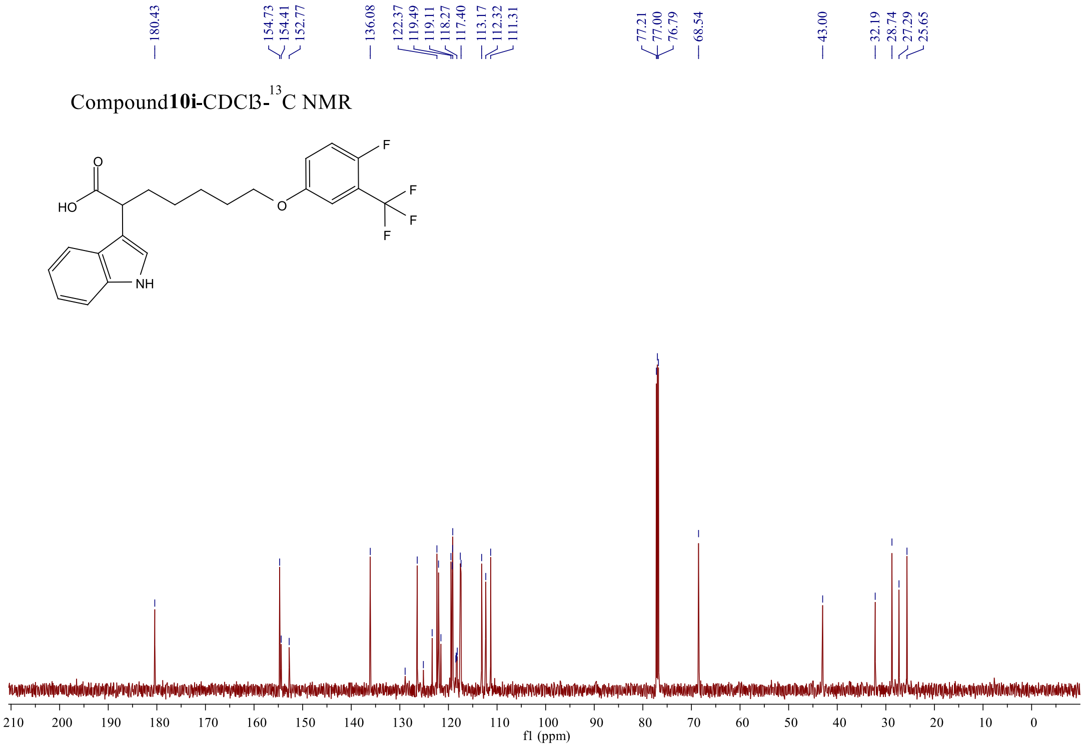

^{13}C NMR spectrum of compound **10i**

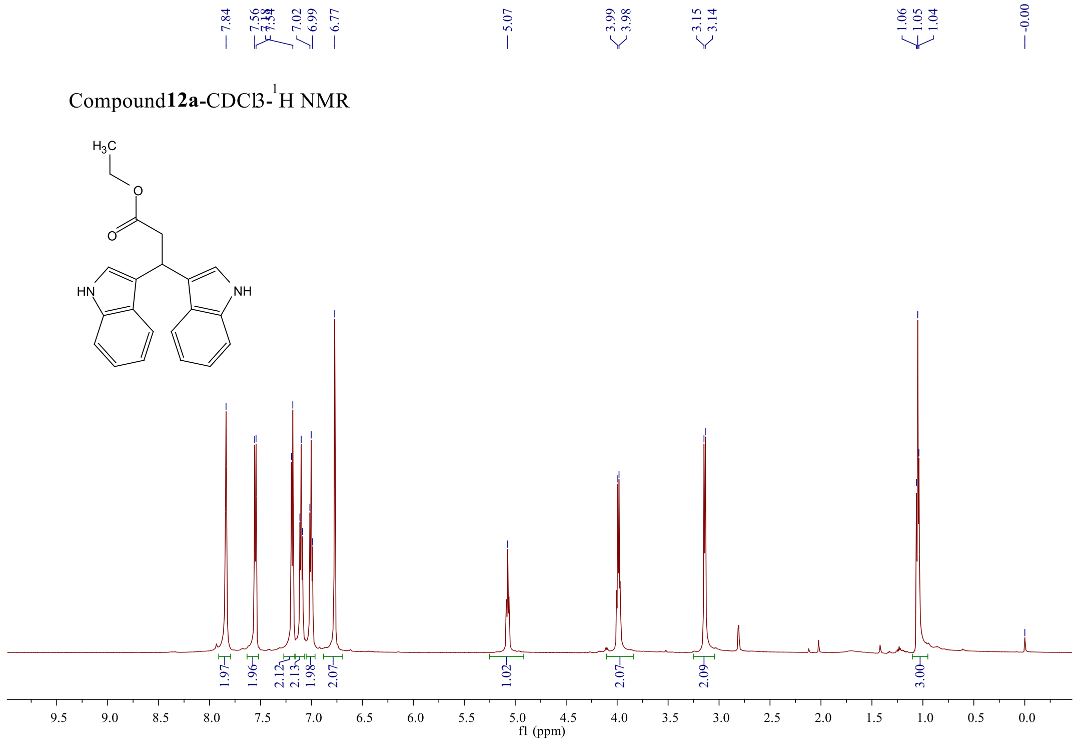

^{1}H NMR spectrum of compound **12a**

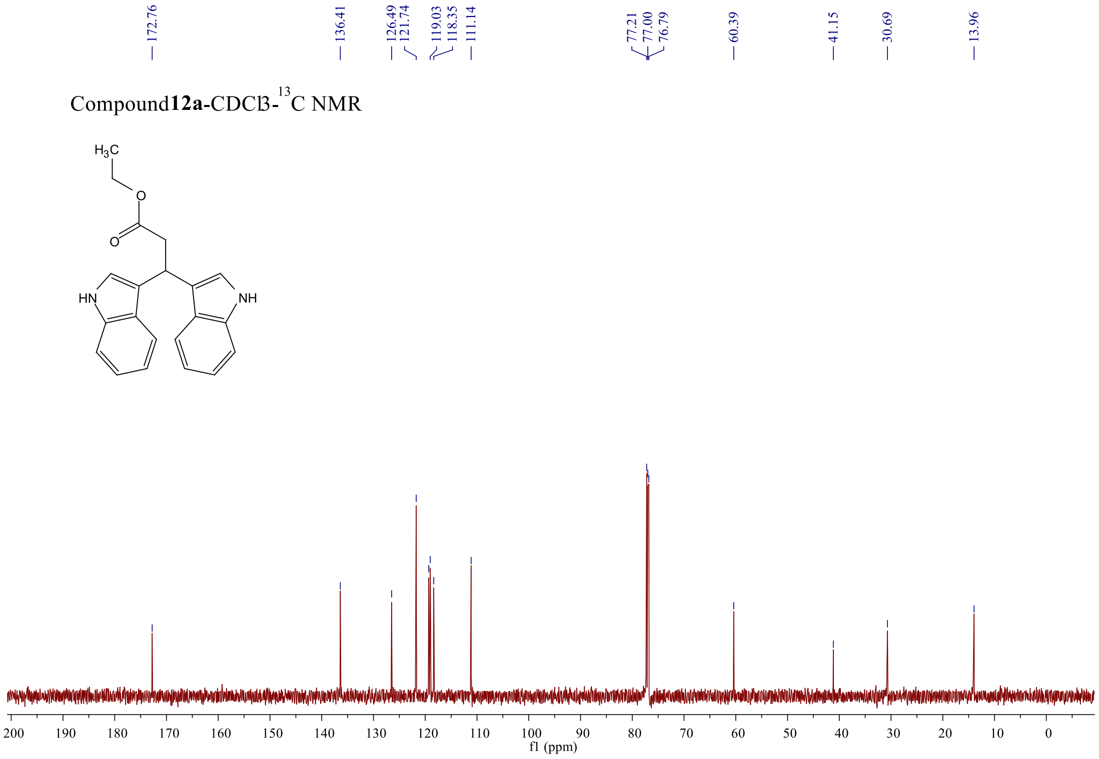

^{13}C NMR spectrum of compound **12a**

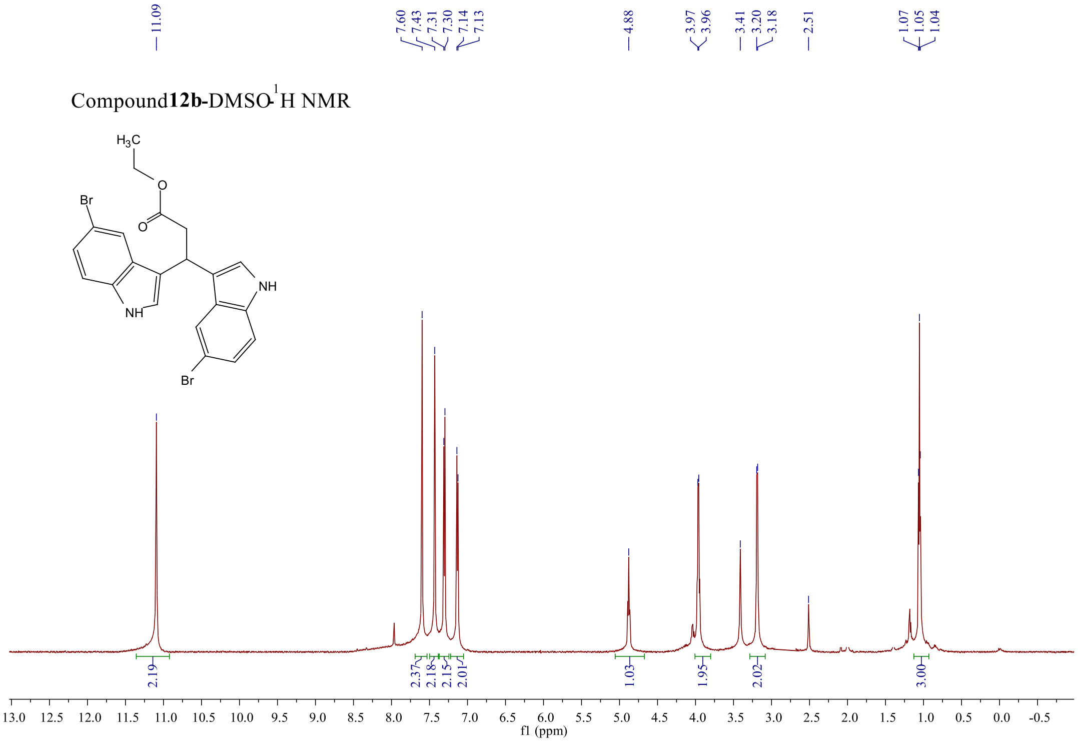

^{1}H NMR spectrum of compound **12b**

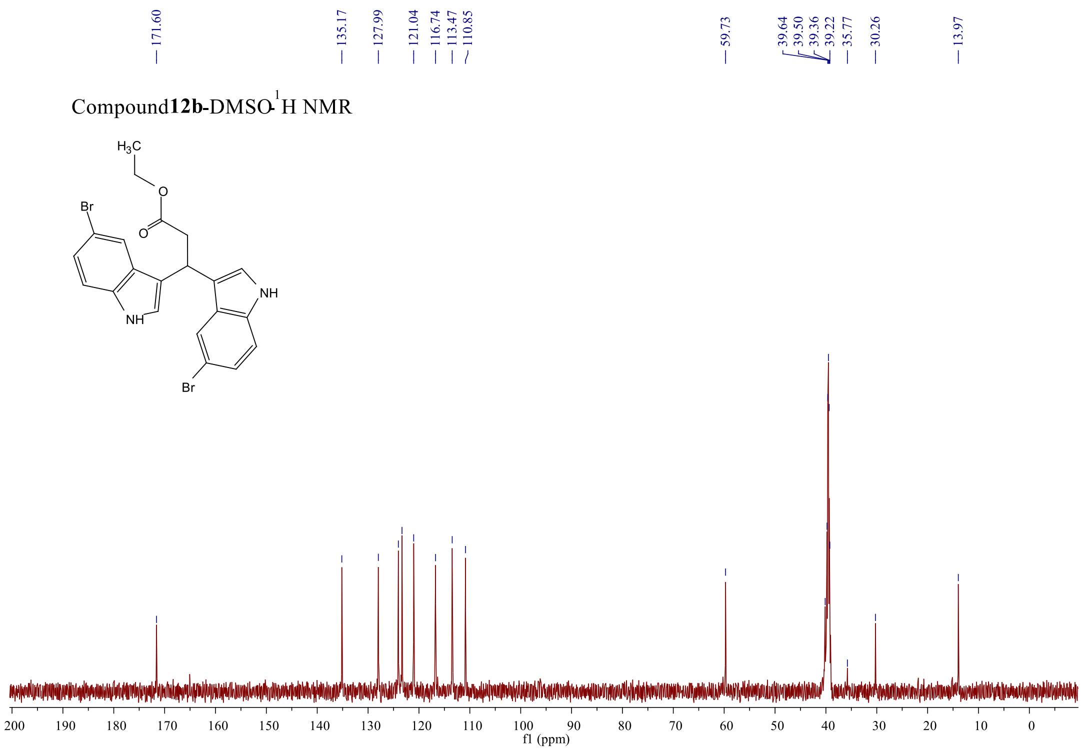

^{13}C NMR spectrum of compound **12b**

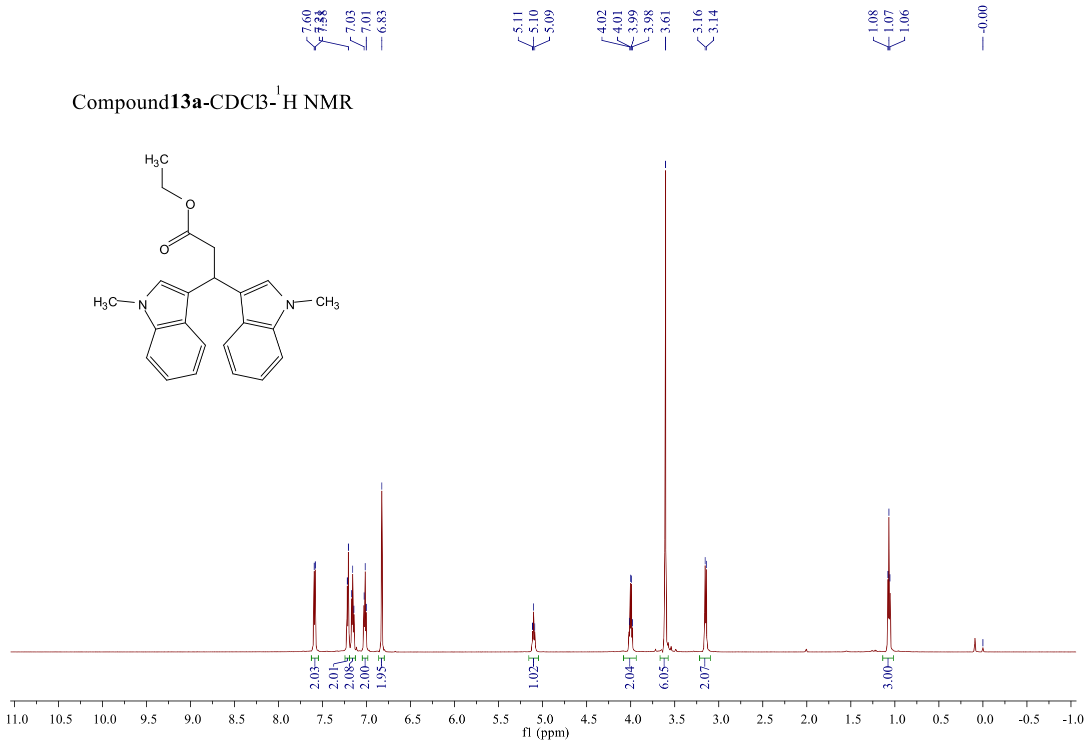

^{1}H NMR spectrum of compound **13a**

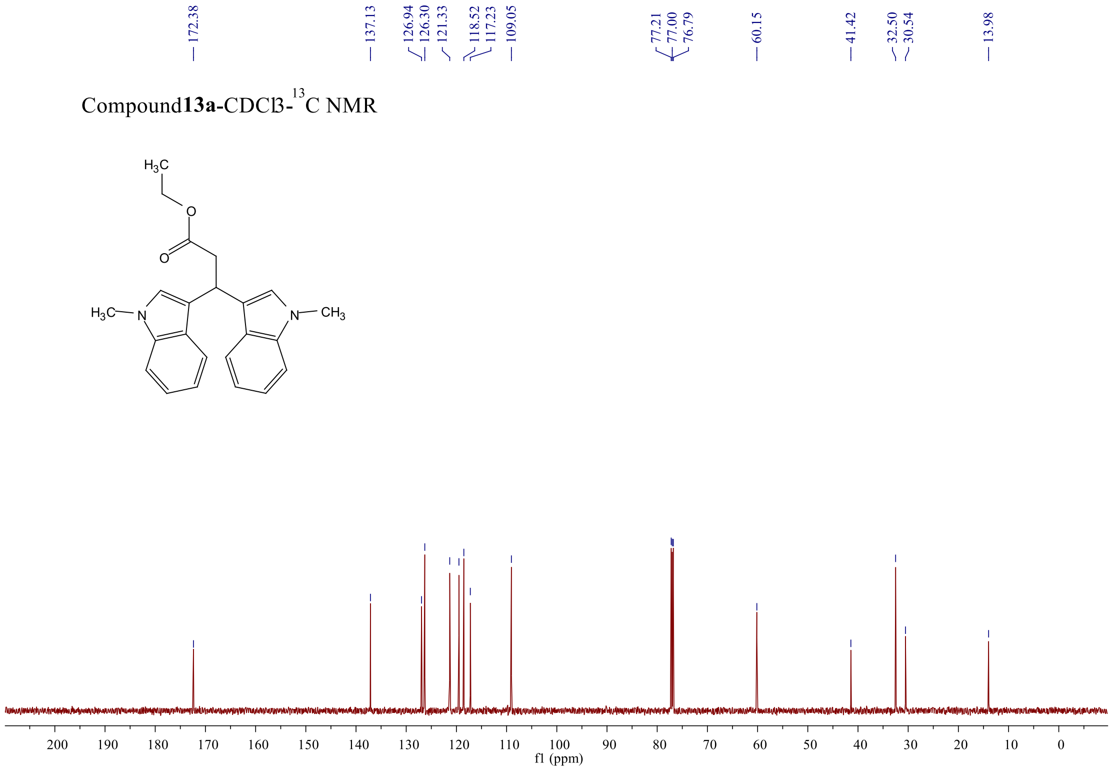

^{13}C NMR spectrum of compound **13a**

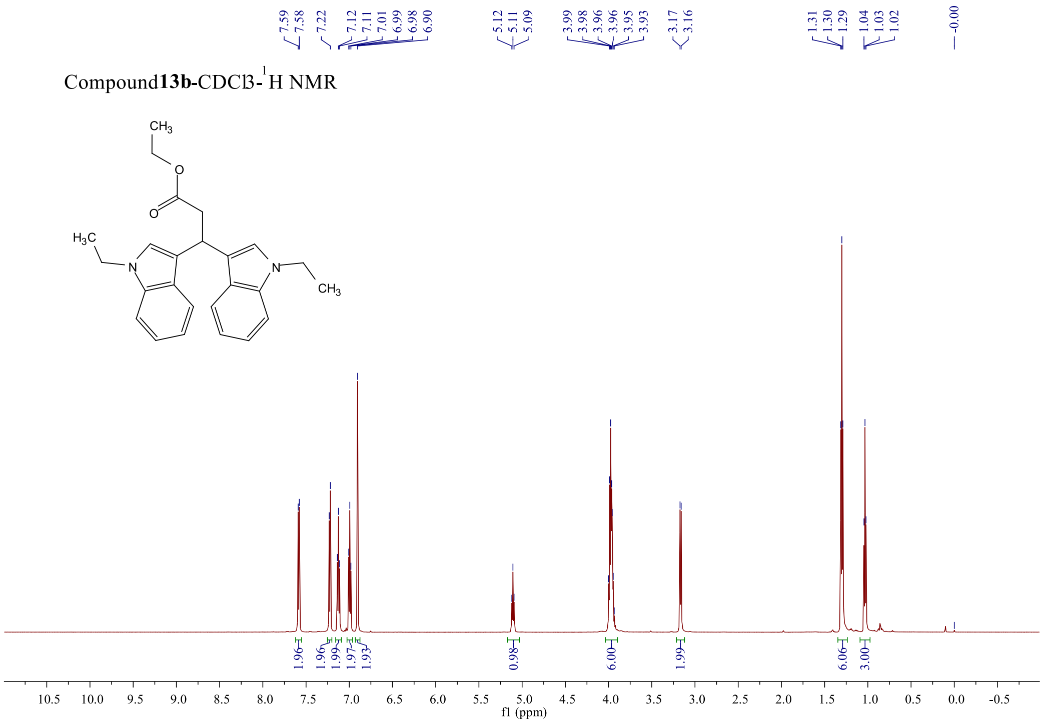

^{1}H NMR spectrum of compound **13b**

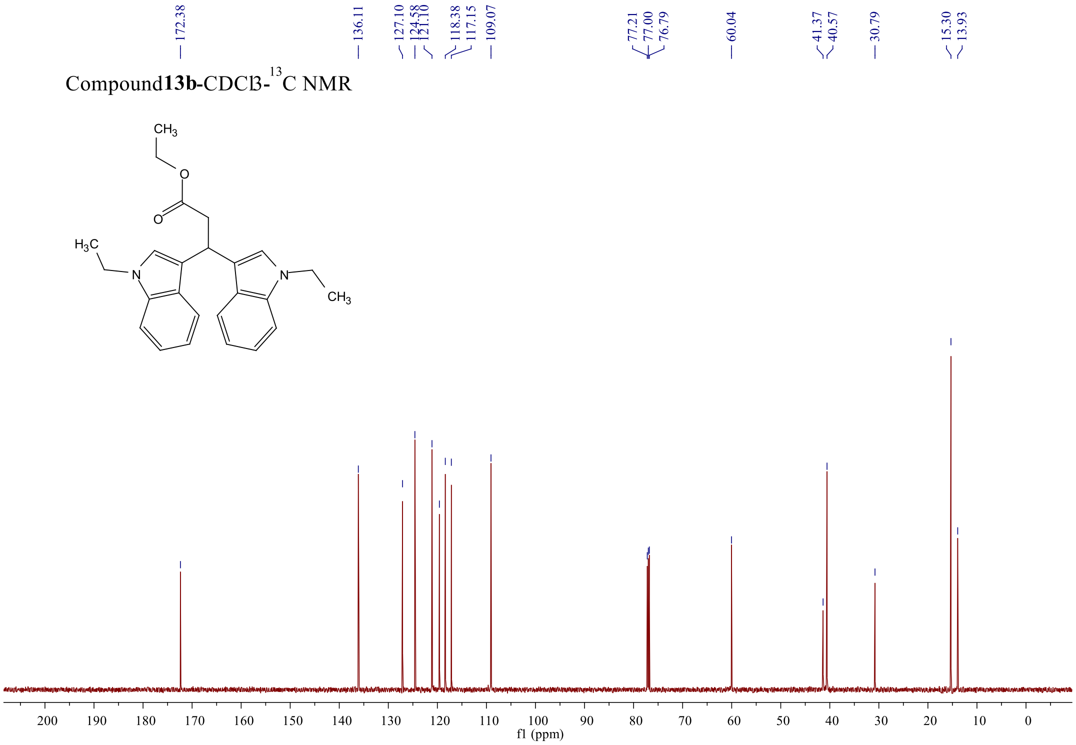

^{13}C NMR spectrum of compound **13b**

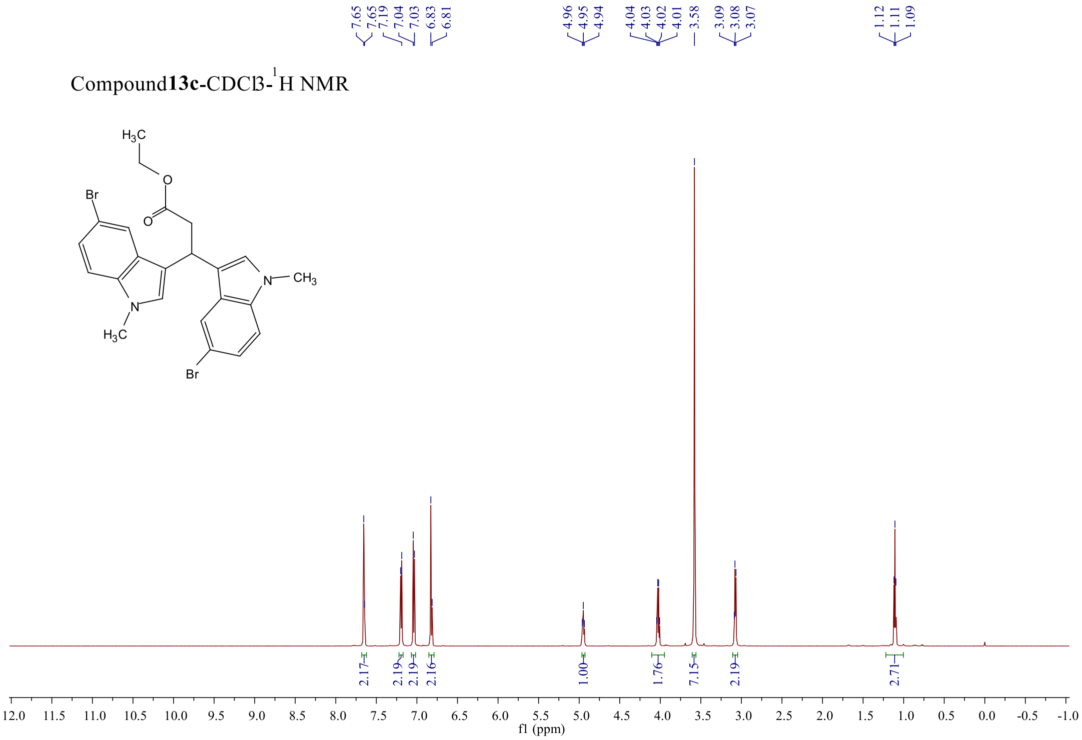

^{1}H NMR spectrum of compound **13c**

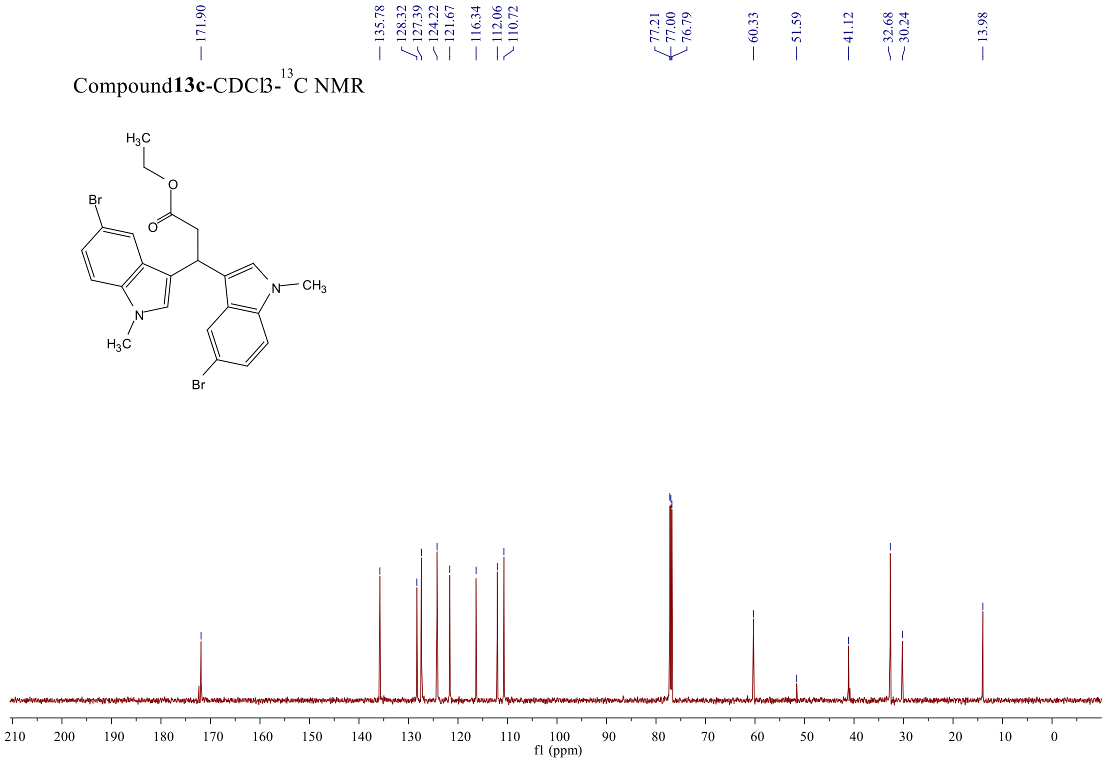

^{13}C NMR spectrum of compound **13c**

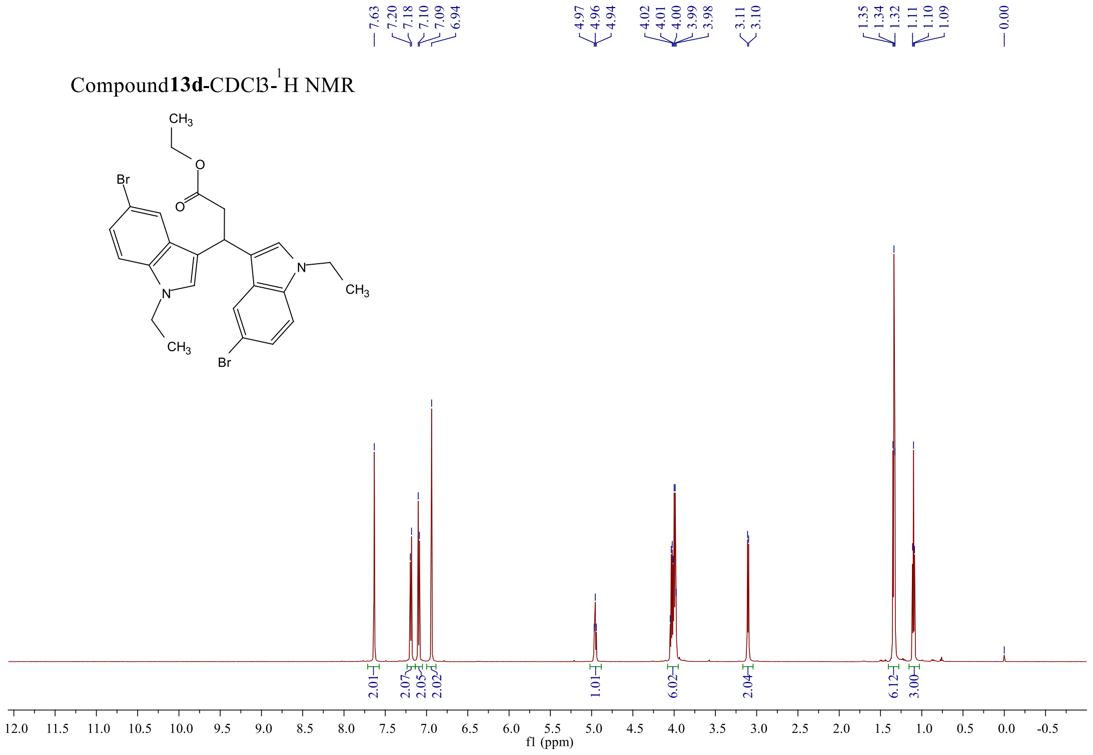

^{1}H NMR spectrum of compound **13d**

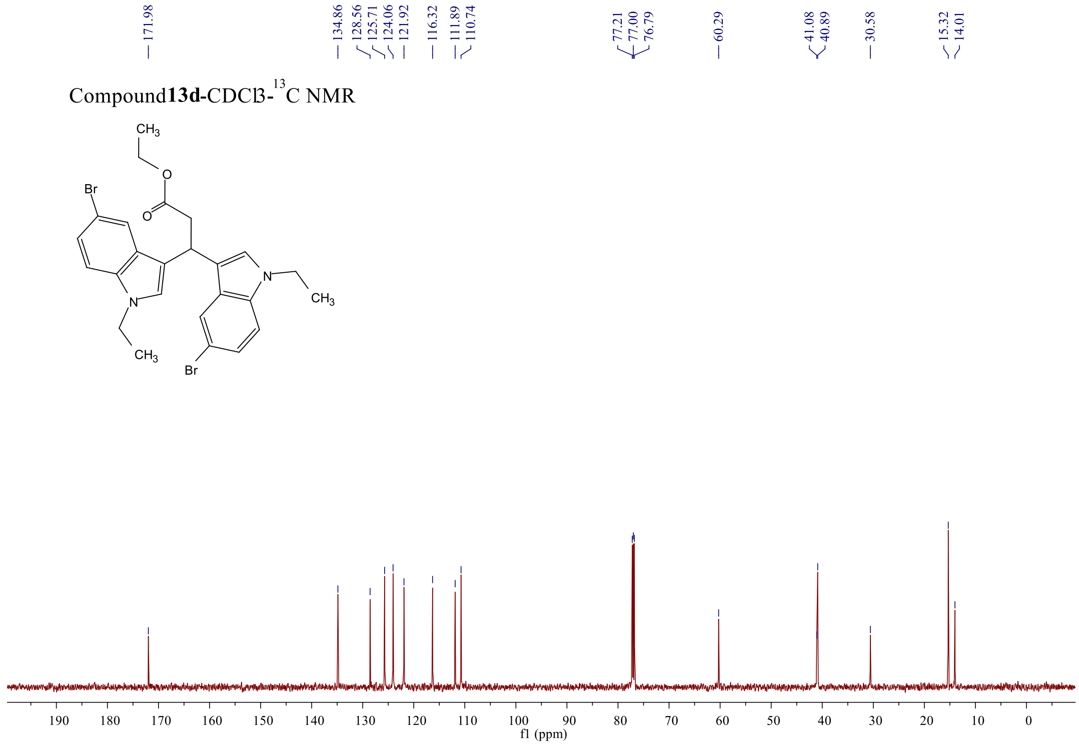

^{13}C NMR spectrum of compound **13d**

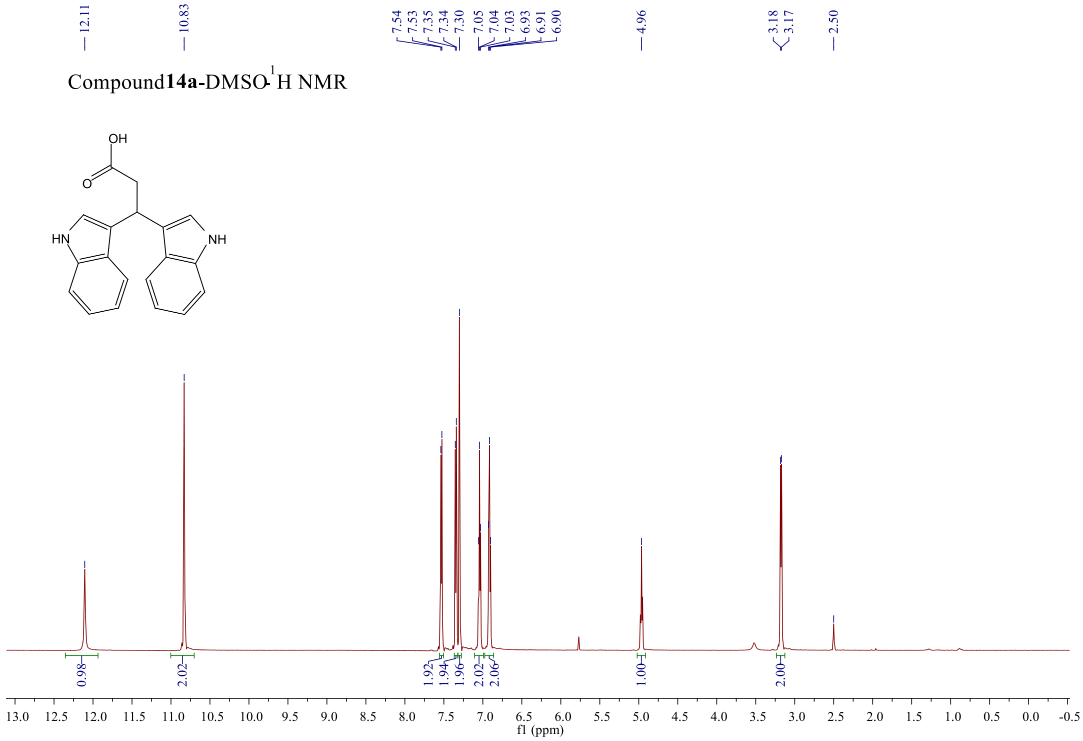

^{1}H NMR spectrum of compound **14a**

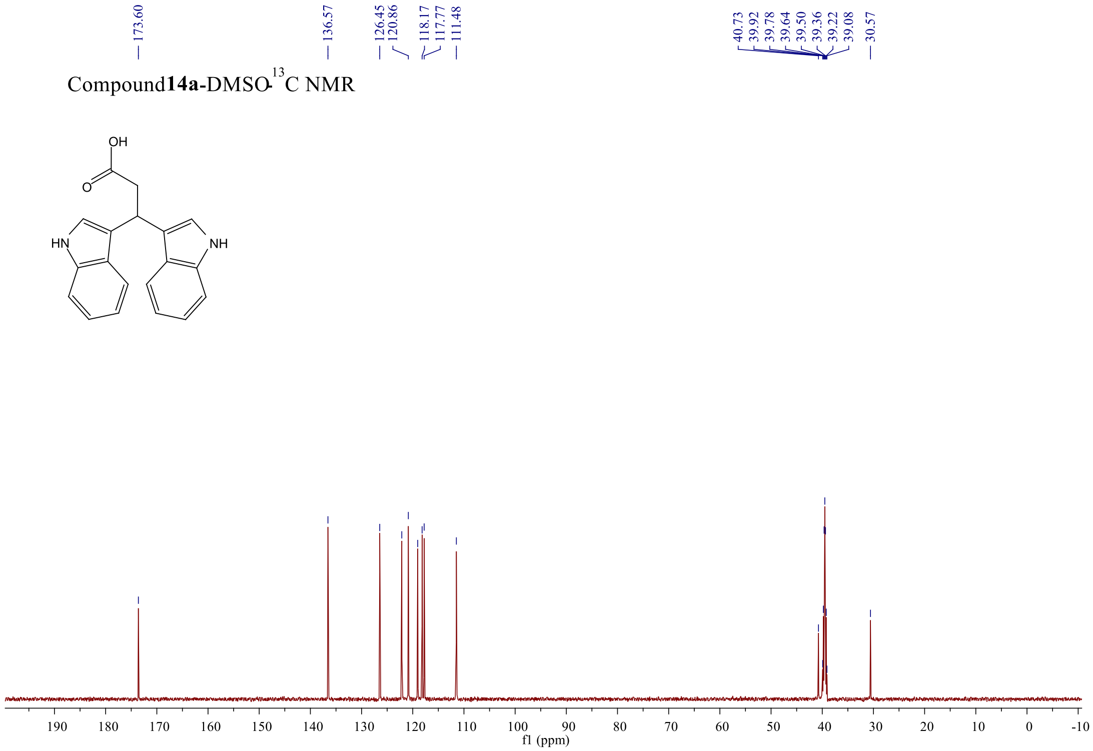

^{13}C NMR spectrum of compound **14a**

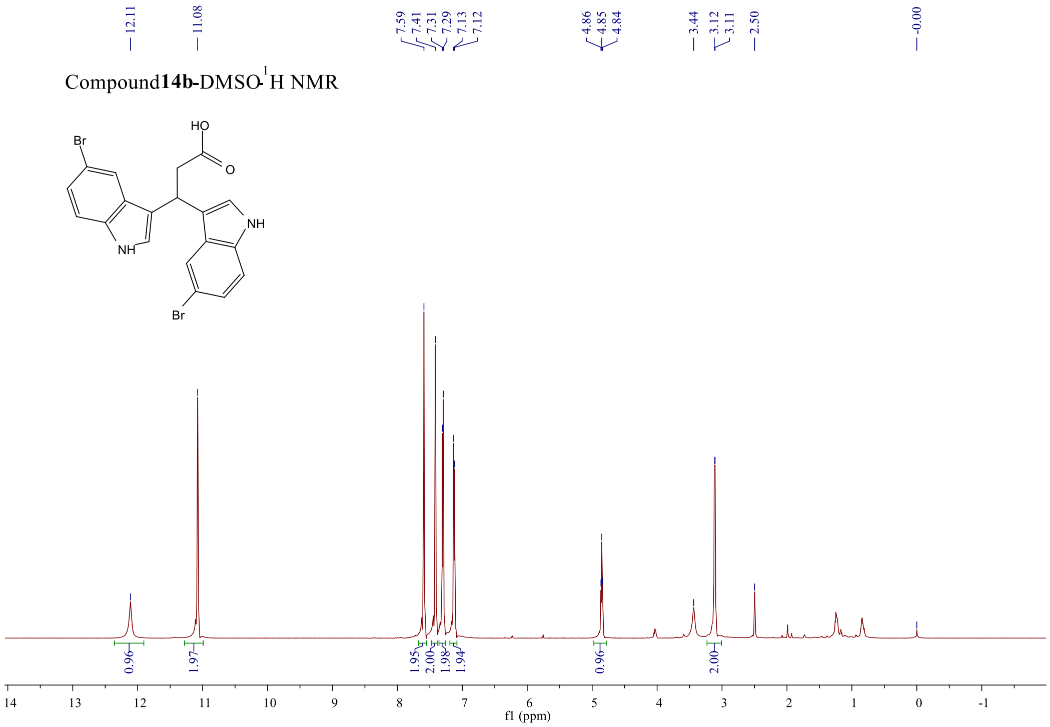

^{1}H NMR spectrum of compound **14b**

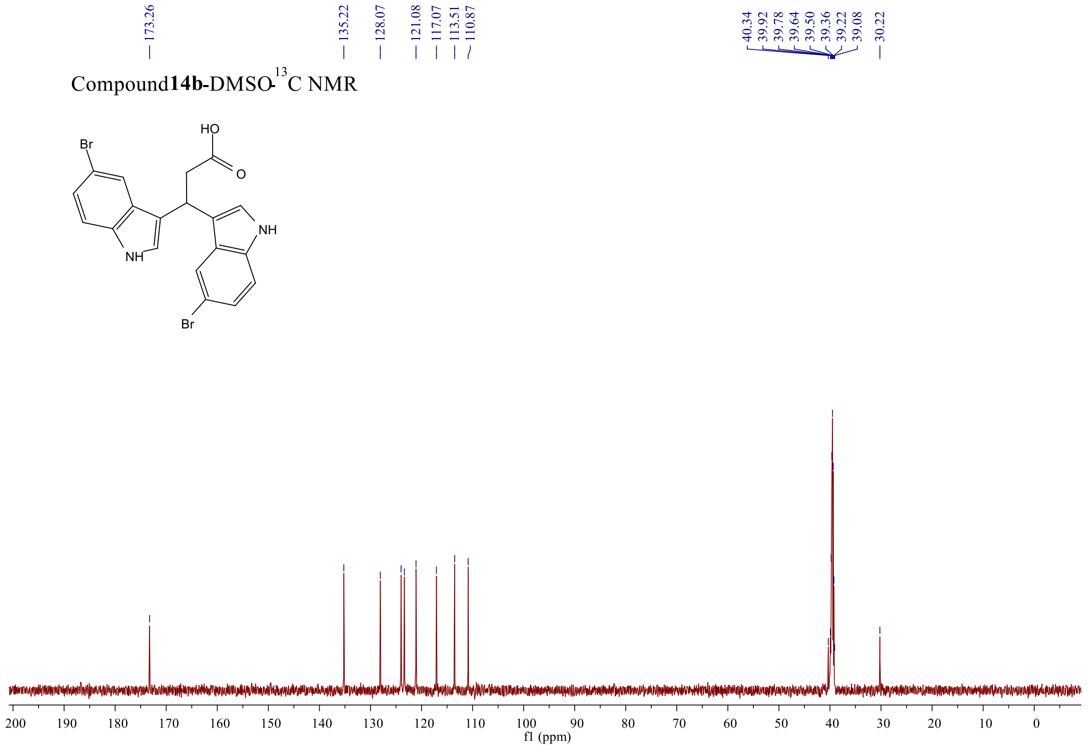

^{13}C NMR spectrum of compound **14b**

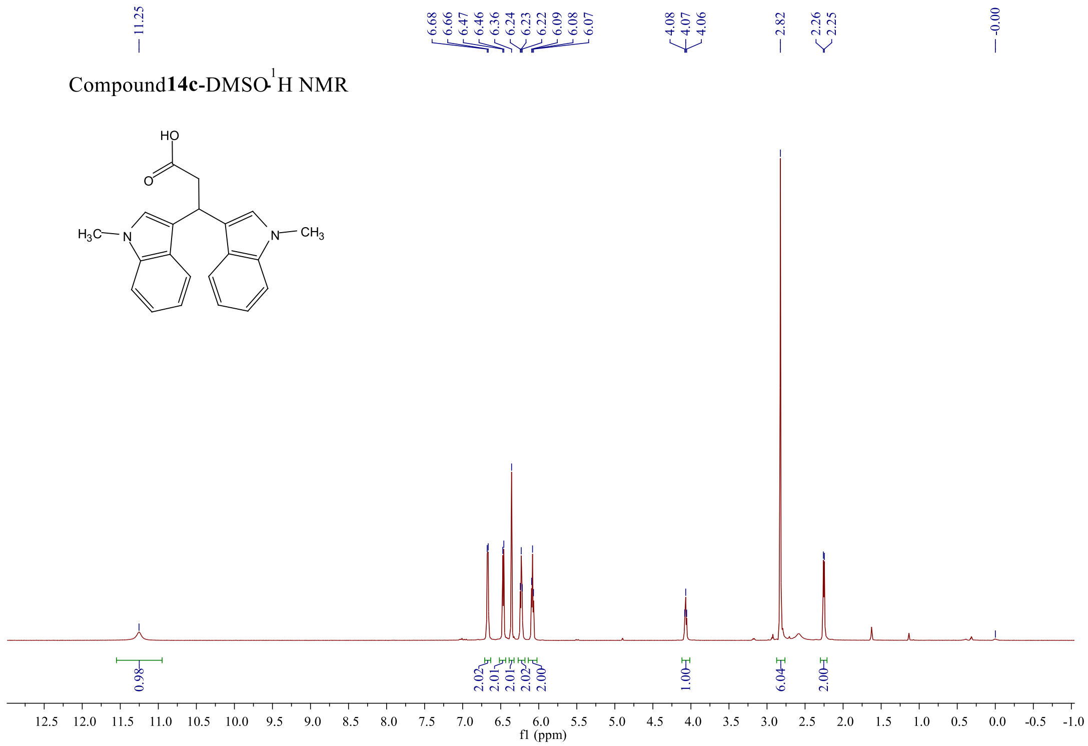

^{1}H NMR spectrum of compound **14c**

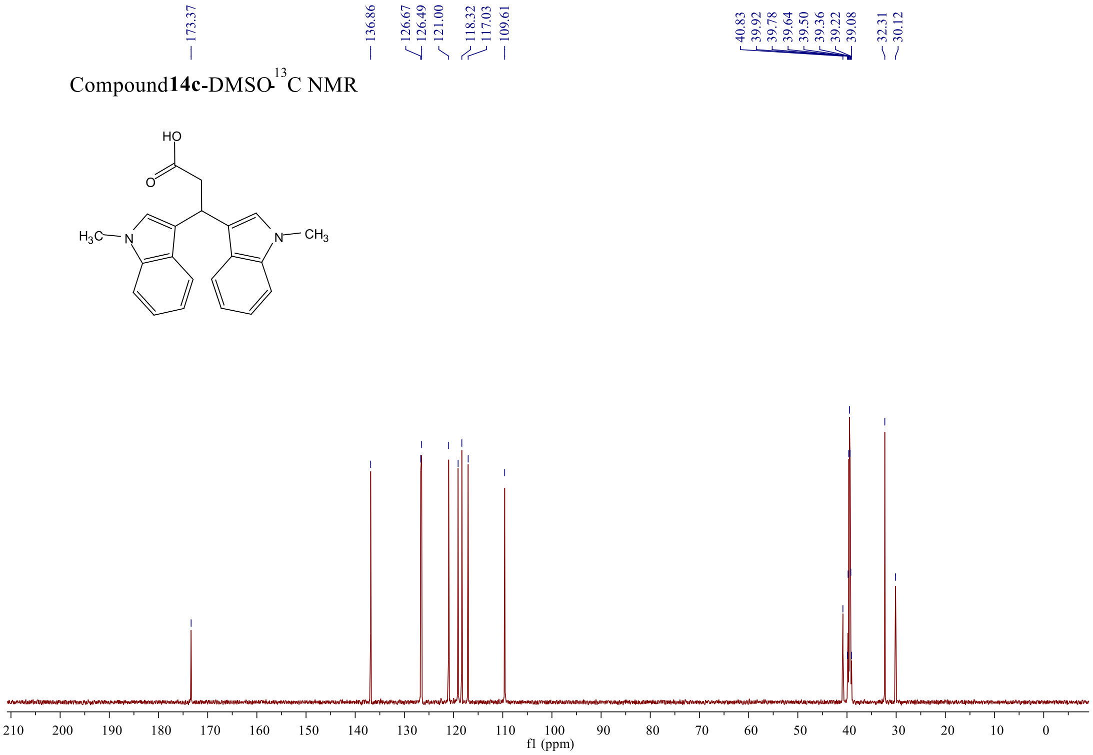

^{13}C NMR spectrum of compound **14c**

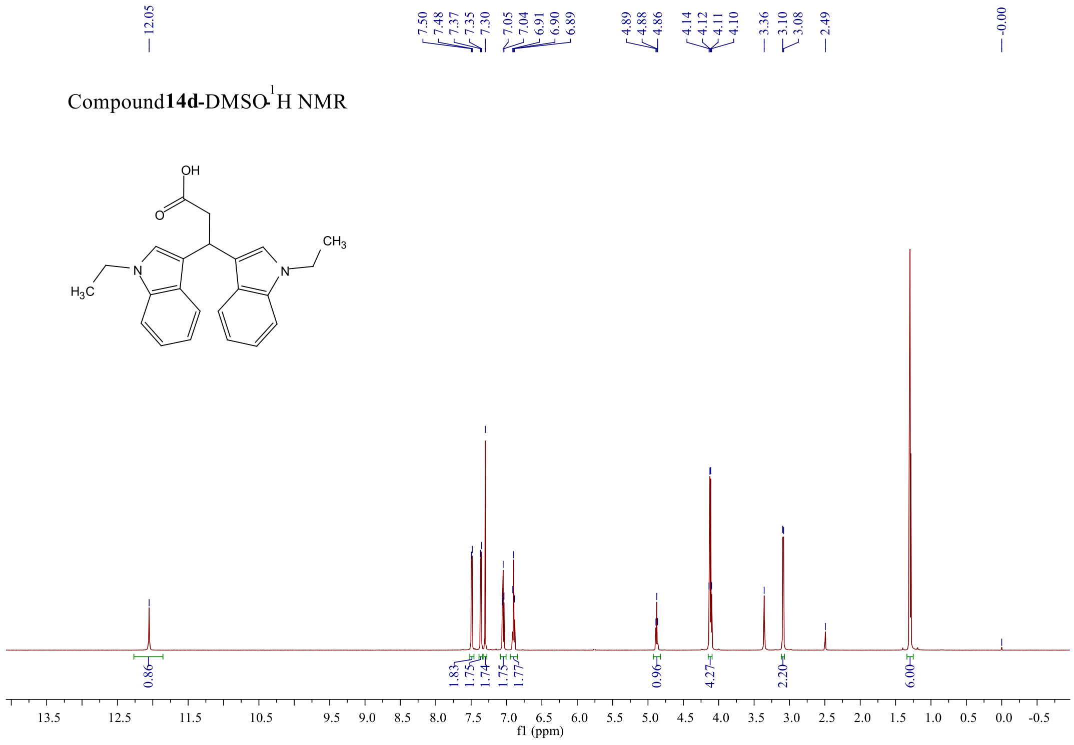

^{1}H NMR spectrum of compound **14d**

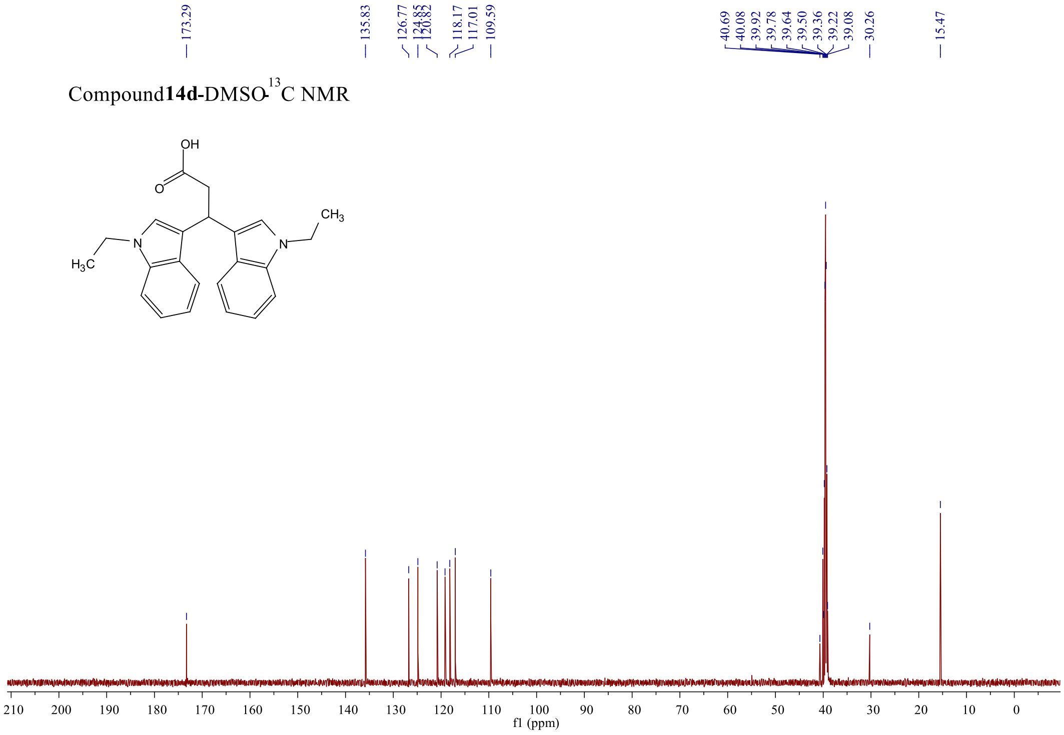

^{13}C NMR spectrum of compound **14d**

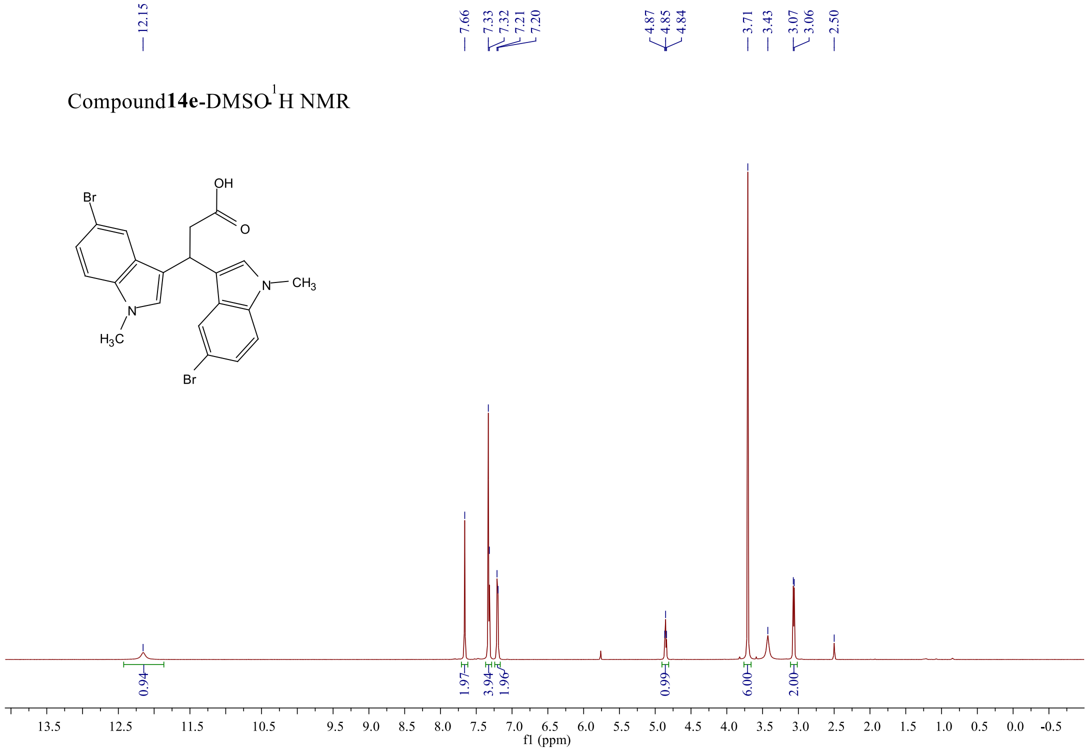

^{1}H NMR spectrum of compound **14e**

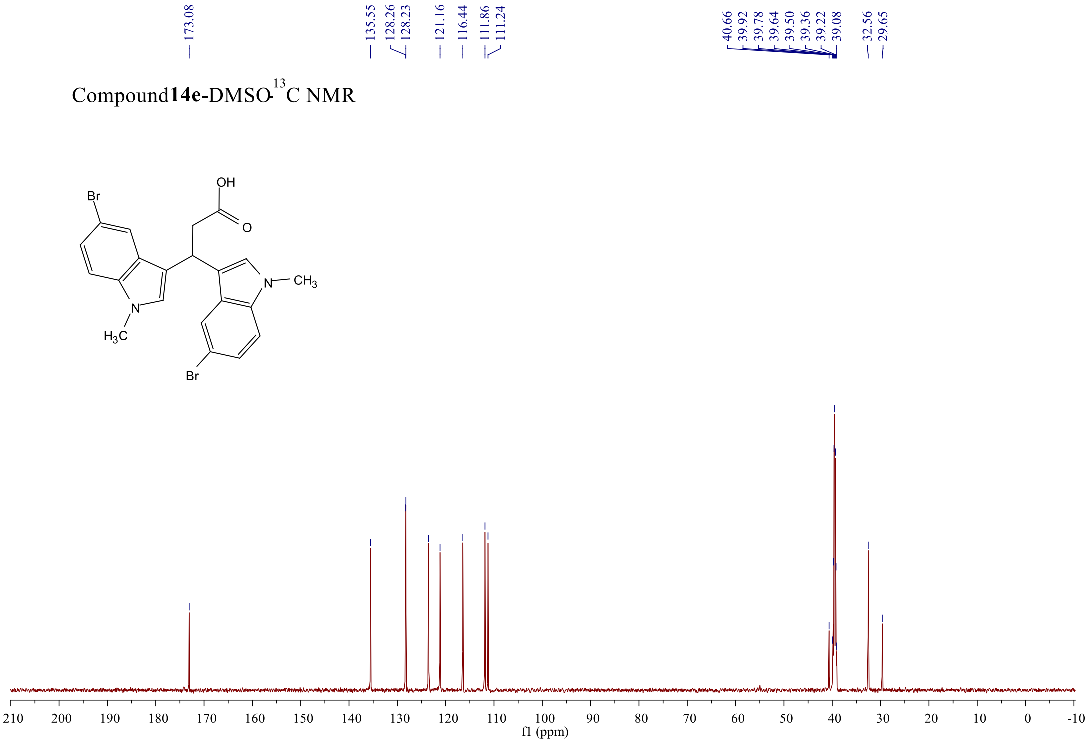

^{13}C NMR spectrum of compound **14e**

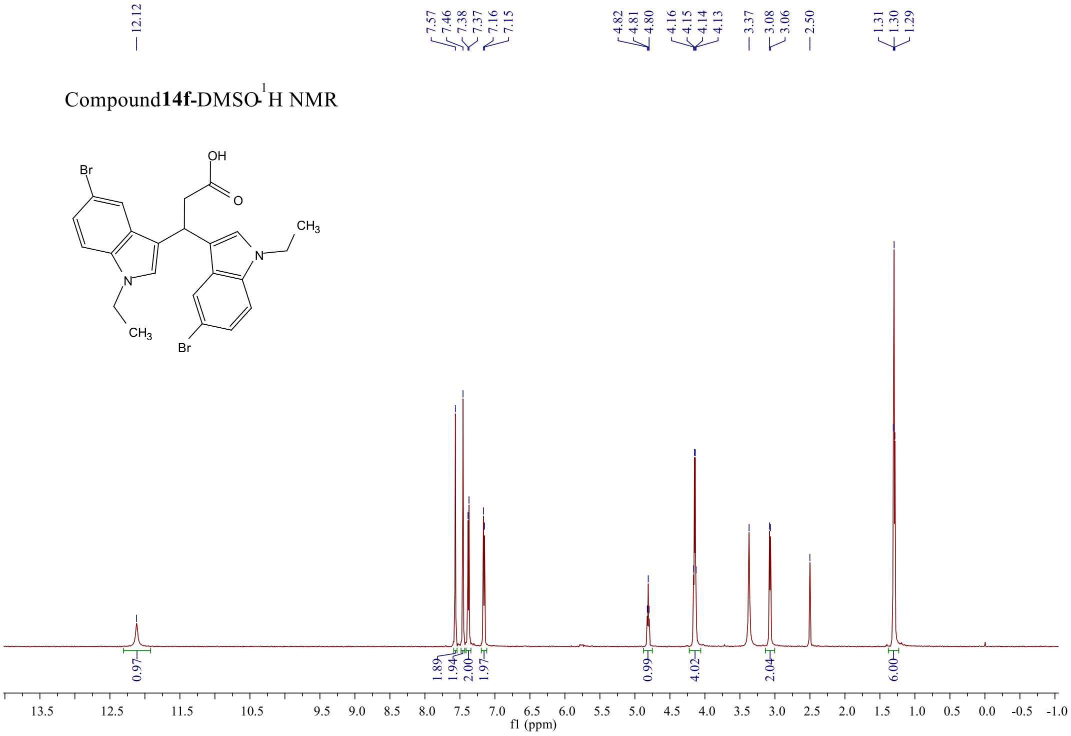

^{1}H NMR spectrum of compound **14f**

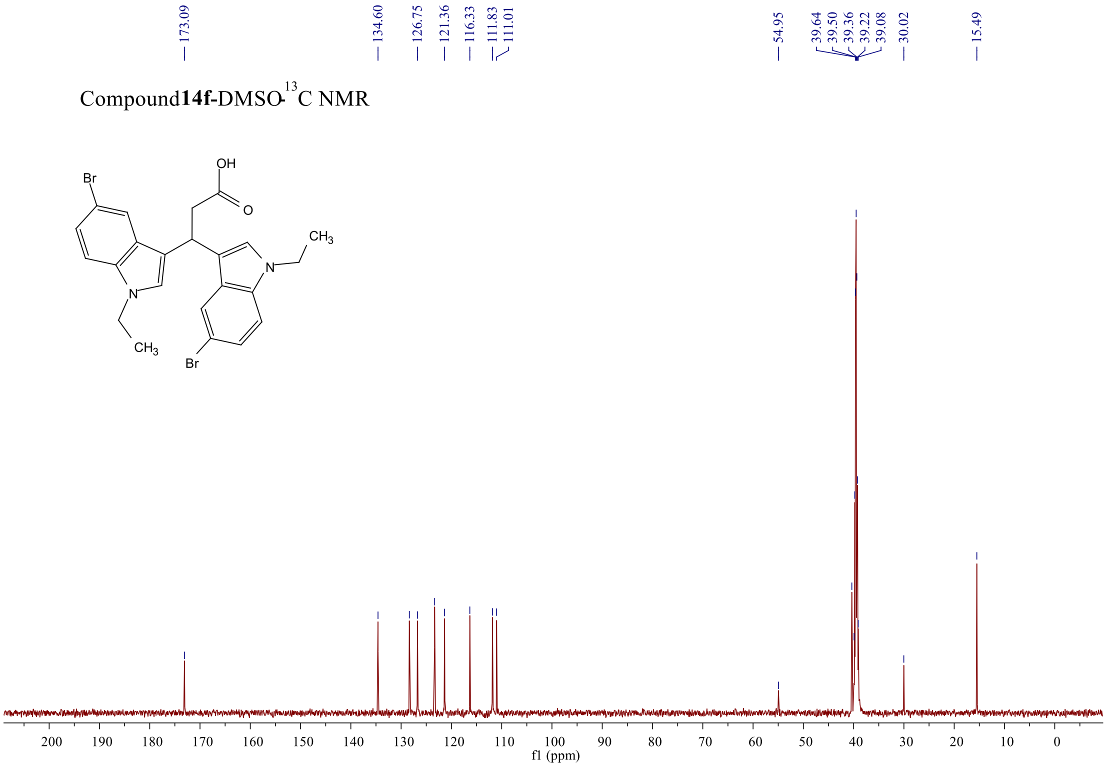

^{13}C NMR spectrum of compound **14f**
